# Transcriptomic analysis of CO_2_-treated strawberries (*Fragaria vesca*) with enhanced resistance to softening and oxidative stress at consumption

**DOI:** 10.3389/fpls.2022.983976

**Published:** 2022-08-19

**Authors:** Ivan del Olmo, Irene Romero, Maria Dolores Alvarez, Rosa Tarradas, Maria Teresa Sanchez-Ballesta, Maria Isabel Escribano, Carmen Merodio

**Affiliations:** Laboratory of Biotechnology and Postharvest Quality, Department of Characterization, Quality and Security, Institute of Food Science, Technology and Nutrition (ICTAN-CSIC), Madrid, Spain

**Keywords:** cell wall, CO_2_, firmness, H_2_O_2_, lignin, RNA-seq, xyloglucans, strawberries

## Abstract

One of the greatest threats to wild strawberries (*Fragaria vesca* Mara des Bois) after harvest is the highly perishability at ambient temperature. Breeders have successfully met the quality demands of consumers, but the prevention of waste after harvest in fleshy fruits is still pending. Most of the waste is due to the accelerated progress of senescence-like process after harvest linked to a rapid loss of water and firmness at ambient temperature. The storage life of strawberries increases at low temperature, but their quality is limited by the loss of cell structure. The application of high CO_2_ concentrations increased firmness during cold storage. However, the key genes related to resistance to softening and cell wall disassembly following transference from cold storage at 20°C remain unclear. Therefore, we performed RNA-seq analysis, constructing a weighted gene co-expression network analysis (WGCNA) to identify which molecular determinants play a role in cell wall integrity, using strawberries with contrasting storage conditions, CO_2_-cold stored (CCS), air-cold stored (ACS), non-cold stored (NCS) kept at ambient temperature, and intact fruit at harvest (AH). The hub genes associated with the cell wall structural architecture of firmer CO_2_-treated strawberries revealed xyloglucans stabilization attributed mainly to a down-regulation of *Csl E1*, *XTH 15*, *Exp-like B1* and the maintenance of expression levels of nucleotide sugars transferases such as *GMP* and *FUT* as well as improved lamella integrity linked to a down-regulation of *RG-lyase*, *PL-like* and *PME*. The preservation of cell wall elasticity together with the up-regulation of *LEA*, *EXPA4*, and *MATE*, required to maintain cell turgor, is the mechanisms controlled by high CO_2_. In stressed air-cold stored strawberries, in addition to an acute softening, there is a preferential transcript accumulation of genes involved in lignin and raffinose pathways. Non-cold stored strawberries kept at 20°C after harvest are characterized by an enrichment in genes mainly involved in oxidative stress and up-expression of genes involved in jasmonate biosynthesis. The present results on transcriptomic analysis of CO_2_-treated strawberries with enhanced resistance to softening and oxidative stress at consumption will help to improve breeding strategies of both wild and cultivated strawberries.

## Highlights

- Gene co-expression network analysis of the transcriptome in CO_2_-treated strawberries identifies genes involved in softening resistance at consumption.
- Identification of molecular determinants underlying the protection against senescence-like process by high CO_2_.
- Oxidative stress in non-cold stored strawberries kept at 20°C after harvest.
- Softening and up-regulation of genes involved in lignin and raffinose pathways in stressed air-cold stored strawberries.
- Preservation of cell wall elasticity, xyloglucans stabilization and improved lamella integrity in CO_2_-treated strawberries.

## Introduction

Strawberries undergo intense breeding selection for new cultivars based on fruit quality traits (Barbey et al., 2020). Breeders have successfully met the demands of consumer preference in taste and nutritional values, but the prevention of waste after harvest in fleshy fruits is still pending. Most of the waste is due to the accelerated progress of senescence-like process after harvest linked to a loss of texture of the fruit. However, genetic studies conducted on texture at consumption after postharvest storage have been limited, partly due to the complexities associated with quantifying it (Cockerton et al., 2021). Hence, understanding of the molecular biology underlying the regulation of fruit texture after harvest is a strategy to tackle eating quality and fruit loss prevention. The softening of strawberries during developmental ripening process is well-studied (Harrison et al., 2001; Park and Cosgrove, 2015; Moya-León et al., 2019; Witasari et al., 2019). Different transgenic strategies have been applied to assess the role of specific genes in controlling fruit softening and in some cases, down-regulating the genes involved in pectin solubilization and depolymerization (Santiago-Doménech et al., 2008; Molina-Hidalgo et al., 2013; Jara et al., 2019). Alternatively, the information gained on wild strawberry genes from the application of beneficial postharvest treatments that enhance firmness may be translated into useful and straightforward ways to improve fruit eating quality. Specifically, wild strawberry *Fragaria vesca* is an important model system for the increased demand of fruit quality traits (Diamanti et al., 2012) in cultivated strawberry because of its diploid genome, availability of extensive transcriptome data, and a range of molecular genetic tools (Hawkins et al., 2017). A problem with wild strawberries is that at ambient temperature they experience a quick softening, fungal attack and water loss. Low temperature storage around 0°C is one of the most important factors to extend storage strawberry life and to delay softening. Although strawberries are classified as not susceptible to chilling injury, they are prone to producing exudate and application of technologies improving water retention is needed. Short-term high CO_2_ treatments help strawberries to face temperature shifts at 0°C (Blanch et al., 2015). In cultivated strawberries, the beneficial effect of high CO_2_ concentrations (15–20% CO_2_) to reduce softening during cold storage is well-known (Harker et al., 2000). The effect of high CO_2_ in the delay of softening during cold storage depends on cultivar., maturity and storage length (Ponce-Valadez et al., 2009), and in some cultivars, including *Fragaria vesca* Mara des Bois, high CO_2_-induced firmness enhancement (Smith and Skog, 1992; Larsen and Watkins, 1995; Harker et al., 2000; Blanch et al., 2019). However, little is known whether the firming effect induced by high CO_2_ concentrations at low temperature was maintained after transfer at 20°C. Since firmness at the time of consumption is crucial to achieve desirable organoleptic characteristics and to reduce waste, it is of importance to understand the effect of high CO_2_ after transference at 20°C following cold storage. Additionally, while the effect of low temperature and short-term high CO_2_ in postharvest storage of berries on transcriptome profiling has been analyzed (Ponce-Valadez et al., 2009; Rosales et al., 2016; Bang et al., 2019) less attention has been paid to the molecular mechanisms in response to the temperature shifts from cold storage at 20°C, which greatly affects fruit quality. Moreover, it cannot be assumed that fruit responses at 20°C after transfer from different cold storage conditions will be identical to that of non-stored fruit, and it is possible that the activation of cold-stress responses to temperature shifts at 0°C might accelerate the loss of firmness at the moment of consumption.

Clearly, the regulation of texture in response to temperature shift at 20°C from cold storage is quite complex and new approaches are needed, including a better understanding of the relationship between changes in the textural properties and senescence-like process. The crucial role of turgor, the force exerted on the cell membrane by intracellular fluid for cell separations in the textural properties of fruit, is well recognized (Toivonen and Brummell, 2008; Castellarin et al., 2016). For the contribution to osmotic adjustment for turgor maintenance and cell wall structure, the metabolism of sugars, especially sucrose, the main reserve carbohydrate in strawberries, takes on particular importance. We previously reported that in the diploid Mara des Bois strawberries, other beneficial responses were found concerning high CO_2_ treatment, primarily sucrose accumulation and water retention (del Olmo et al., 2020).

The aim of the present work was firstly to determine which molecular mechanisms participate in senescence-like responses and degradative process controlled by high CO_2_ levels. Secondly, to identify the key genes related to resistance to softening and cell wall disassembly following transference from cold storage at 20°C. For this, we undertook a transcriptomic approach on Mara des Bois strawberries with storage conditions, with CO_2_-cold stored (CCS), air-cold stored (ACS), non-cold stored (NCS) and intact fruit at harvest (AH) acting as quality control samples. We applied weight gene co-expression network analysis (WGCNA) to explore candidate biomarkers of firmness from the perspective of a weighted network. Furthermore, generating the whole genome transcriptome of *Fragaria vesca* serves as a powerful tool for unraveling cross-talk molecular mechanisms of softening and senescence, which may have negative impacts on quality of wild and cultivated strawberries.

## Materials and methods

### Plant material

Mara des Bois strawberries (*Fragaria vesca*) were harvested from a commercial crop in San Sebastian de los Reyes (Madrid, Spain). The ripe strawberries immediately after harvest (2 h) were used as the control (AH). 40 boxes of 200 g of fruit placed directly in a chamber at 20°C and 80% RH for 2 days were the non-cold stored (NCS). Another group of 80 boxes of fruit were randomly divided into two lots and stored in two containers at 1°C. One container was stored in air for 7 days and then transferred at 20°C for 1 day (conditions employed to simulate a time in transit) and used to analyze the air-cold stored fruit (ACS). The other container was pretreated for 2 days with a gas mixture containing 18% CO_2_ + 18% O_2_ + 64% N_2_, then air-ventilated for another 5 days and thereafter transferred at 20°C for 1 day and used to analyze the CO_2_-cold stored fruit (CCS). Five boxes (90 strawberries) from each storage condition (control, non-cold stored, air-cold stored and CO_2_-cold stored) were collected. 45 strawberries were assessed for texture and quality, while another 45 were divided into three batches of 15 berries and frozen in liquid nitrogen and stored at-80°C until analysis.

### Glutathione, lignin, H_2_O_2_ and major soluble sugars

Glutation content was determined following the extraction of 100 mg of frozen strawberry in 0.5 ml of 5% 5-sulfosalicylic acid (SSA), After centrifugation at 8000 x g for 10 min, the supernatant was recovered and diluted (1:10) with ddH_2_O. Glutathione content (GSH + GSSG, reduced plus oxidized glutathione forms) was established by using the commercial kit from Sigma-Aldrich, with the change in absorbance at 405 nm recorded over 10 min using a BioTek PowerWave XS microplate reader (BioTek, France). Glutathione concentrations were extrapolated from standard curves of GSSG *y* = 0.0366*x*–0.0121; *R*^2^ = 0.9988 and Glutathione total *y* = 0.0251*x*–0.0159; *R*^2^ = 0.9997.

Lignin content was determined using activated triethylene glycol (TEG) with modifications (Edwards, 1973). One L of TEG was activated with 6.3 ml of HCl (37%). One g of frozen strawberry powder was suspended in 7 ml of activated TEG and left for 1 h at 121°C. The samples were centrifuged at 4,000 rpm for 10 min, and the supernatant was recovered. The residue was extracted a second time by adding 7 ml of activated TEG, agitated in vortex and centrifugated at 4,000 rpm for 10 min. The supernatants were combined and diluted at 1:50, and their absorbance was measured at 280 nm using a BioTek PowerWave XS microplate reader (BioTek, France). A calibration curve was prepared in the concentration range from 0 to 100 mg l^−1^ of lignin (Sigma-Aldrich; *y* = 0.0284*x*–0.0216; *R*^2^ = 0.9931).

Hydrogen peroxide was measured photometrically after reaction with KI. 500 mg of frozen strawberry powder was suspended in 1.5 ml of ice-cold 0.1% TCA. The samples were centrifuged at 12,000 rpm for 15 min at 4°C and the supernatant was recovered. The reaction mixture consisted of 0.2 ml supernatant, 0.2 ml H_2_O, 12 μl EDTA 10 mM, and 0.8 ml KI 1 M. The reaction was developed for 30 min in darkness and absorbance measured at 390 nm using a BioTek PowerWave XS microplate reader (BioTek, France). A calibration curve of H_2_O_2_ (Sigma-Aldrich) from 0 to 150 μM was prepared (*y* = 0.0011*x*–0.0015; *R*^2^ = 0.9997). For sugar analyses, 2 g of frozen strawberry powder (wet) were extracted with 5 ml of distilled water and soluble glucose, fructose, sucrose and xylose were determined as described by del Olmo et al. (2020). Experimental data represent the mean and SD of the three replicates. Each biological replicate was composed of 15 pooled strawberries.

### Texture measurement

Strawberry firmness was analyzed using a TA.HDPlus Texture Analyzer (Stable Micro Systems, Ltd., Godalming, United Kingdom) provided with Texture Exponent software (version 6.1.13.0) and equipped with a 30 kg load cell. Volodkevich tests were carried out using the upper Volodkevich Bite Jaw probe, which performs an imitative test by simulating the action of an incisor tooth biting through the strawberry. Each fruit was placed on the stationary plate, and the biting action was given by the compressive movement of the upper jaw shearing into the equator of the sample to a curve, from which we obtained the first force peak (*N*), the average shearing force (*N*), and the shearing energy (*J*).

### RNA extraction and quality assessment

The total RNA of three biological replicate samples was extracted from 0.5 g of fruit powder in accordance with Yu et al. (2012). Each biological replicate sample contained a fruit mixture of at least 50 strawberries. A TURBO DNase™ enzyme (Ambion, Austin, TX, United States) treatment was used to degrade the contaminant DNA. RNA quantity and purity were measured with the NanoDrop ND-1000 spectrophotometer (Thermo Scientific). RNA integrity was determined by the RNA integrity number (RIN), using a 2,100 Bioanalyzer (Agilent) at the CNAG-CRG (Centro Nacional de Análisis Genómico, Barcelona, Spain).

### RNA library construction and RNA sequencing

Samples were sequenced at the CNAG-CRG. The mRNA from each biological replica was employed to prepare the RNA-Seq libraries with the reagents provided in the Illumina^®^TruSeq^™^ Stranded Total RNA kit protocol (Illumina Inc., San Diego, CA, United States). The size and quality of the libraries were quality controlled in Agilent DNA 2100 Bioanalyzer assay (Agilent).

Each library was sequenced using Illumina HiSeq™ 2,500 (Illumina Inc.), in paired-end mode with a read length of 2 × 50 bp, generating minimally 39 million paired-end reads per replica and passing filter for each RNA-Seq library in a fraction of a sequencing lane of the sequencer following the manufacturer’s protocol (max. 200 M/lane). We obtained the following million paired-end reads in each replica: 45, 47, and 64 for AH; 39, 34, and 50 for SL; 39, 45, and 57 for ACS; and 41, 45, and 51 for CCS.

Image analysis, base calling, and base quality scoring of the run were processed by Sequencer Software HiSeq Control Software 2.2.58—Real-Time Analysis (RTA 1.13.48), followed by the generation of FASTQ sequence files by CASAVA 1.8. The base quality of the sequences obtained was also checked with the FastQC tool,^1^ and SeqMonk Mapped Sequence Data Analyzer (version: 1.46.0; Babraham Bioinformatics, United Kingdom). Likewise, FASTQ Groomer (Version 1.1.5) and Cutadapt (Version 1.16.6) were used to optimize the reads, converting FASTQ quality formats and removing adapter sequences.

### Sequence alignment with the reference genome

The aligned files were generated with the HISAT 2.1.0 program (Langmead et al., 2009) that was employed for aligning the sequences against *Fragaria vesca* Whole Genome v4.0.a2 (Li et al., 2019).

### Differential expression analysis

DESeq2 was used to identify differentially expressed genes (DEGs) based on the negative binomial distribution that is generalized for linear models (Love et al., 2014). This program allows us to select our triplicates with at least two data stores in each and will identify probes whose representation differs significantly in the two sets. The RNA-Seq quantitation pipeline tool of SeqMonk Mapped Sequence Data Analyzer (version: 1.46.0; Babraham Bioinformatics, United Kingdom) was used to analyze RNA-Seq expression data. The DESeq2 stats filter was applied in all probes where each comparison (AH vs. SL, AH vs. ACS, AH vs. CCS, NCS vs. ACS, NCS vs. CCS, ACS vs. CCS) had a significance below 0.05 after Benjamini and Hochberg correction was used with independent intensity filtering. Quantitation was performed through RNA-Seq pipeline quantitation counting reads over exons as raw counts, assuming a non-strand specific library. Therefore, each DEGs of each comparison yielded the following number from the 34,008 total probes: (6,167, 7,587, 6,831, 7,796, 7,876, and 3,900 probes, respectively). Finally, the DEGs were delimited based on an absolute log_2_-fold change (Fc) value ≥1.5 for activated genes and ≤ −1.5 for repressed genes which in turn have a False Discovery Rate ≤ 0.05 (FDR ≤ 0.05). Venn diagrams comparing the different sets of DEGs were plotted with the Venn Diagram Plotter.^2^

### Weighted gene co-expression network analysis

Co-expression network analysis was performed using the WGCNA package in R (Version: 1.68; Langfelder and Horvath, 2008). The raw data was prepared before running the WGCNA package by carrying out the following steps. The raw counts for 34,008 genes were normalized on Reads Per Kilobase Million (RPKM). Transcript features over each gene were performed using SeqMonk Mapped Sequence Data Analyzer (version: 1.46.0; Babraham Bioinformatics, United Kingdom). Thus, the RPKM quantification for existing probes was performed with the Read Count Quantitation, using all reads corrected for total count per million reads, which were then corrected by the probe length. The normalized count file was adjusted to match the format that the WGCNA package needed, checking gene outliers and removing them until the cuts were passed. Next, we examined the sample network based on squared Euclidean. The whole network connectivity was calculated, with samples designated as outlying if their Z.k value was below the threshold (thresholdZ.k = −2.5). Therefore, the outlying samples were removed from expression and trait data, and a set of soft-thresholding powers of 20, powers = *c* (1:20), were selected. We were then able to use the pickSoftThreshold function that analyses network topology to choose a proper soft-thresholding power (sft, power = 8). Firstly, we employed automatic module detection *via* dynamic tree cutting, where the function blockwiseModules automatically implements all steps of module detection. Afterward, we calculated a one-step network and defined the gene significance variable (GS.value) for each module color. An intramodular analysis was then carried out, identifying genes with high GS and the color member module, and calculating the gene relationship to trait and important modules. In this way, the gene significance (GS) and module (kME) correlation for each module color were obtained. We also performed stepwise manual module detection to represent the relationships between the modules and trait. Thus, we precisely determined which color module should be selected, defining a dissimilarity based on the topological overlap with the dissTOM = TOMdist (A) function. The cutting method for selecting the module is detailed in Langfelder et al. (2008). Finally, in order to construct and analyze a network with such a large number of nodes, we used the “bwnet” function to construct a network with the blockwiseModules function calculating the topographical overlap matrix (TOM). Finally, the network was visualized using Cytoscape_v3.7.2.

### Functional annotation of unigenes and annotation enrichment analysis

We used the ontological annotations described in The Genome Database for Rosaceae (GDR), which comprises about 52% of all *Fragaria vesca* genome annotations, and enriched them with those obtained in similarity sequence analysis from *Arabidopsis* ontology databases (23% of the annotations) and UNIPROT databases (13% of the annotations). Thus, it was possible to cover 88% of ontologies from the total (≈ 30,000 ORF) of the annotations of *Fragaria vesca* genome.

A Singular Enrichment Analysis (SEA; Du et al., 2010) was performed to iteratively test the functional category functionally enriched (FDR ≤ 0.05) deemed as being enriched and therefore representative of each condition. These ontological analyses have been represented schematically, using descriptions that include the GO categories collected in databases with tools such as REVIGO (Supek et al., 2011), making it possible to visualize long lists of Genetic Ontology terms.

### Validation of differentially expressed genes by RT-PCR

The cDNA was prepared by reverse transcription of 1 μg of total RNA using the Maxima cDNA Kit with the dsDNase kit (Thermo Fisher Scientific, Waltham, MA, United States) following the manufacturer’s instructions. Quantification was performed by real-time quantitative RT-PCR (qPCR) using iCycler iQ^™^ Real-Time PCR Detection System (BIORAD) and quantified using Real-Time Detection System Software (version 2.0). The amplification reactions were carried out in a final volume of 12 μl containing 6 μl of NZY qPCR Green Master Mix (2×; NZYTech, Ltd), 1 μl of each primer (10 μM), and 1 μl of the cDNA. The PCR profile used was 2 min at 50°C, 95°C for 10 min, followed by 40 cycles of 20 s at 95°C and 30 s at 55 or 60°C. Three technical replicates were made from each of the genes studied. Gene expression was determined by the 2^−ΔΔCT^ method using the *F. vesca* Actin-97-like (*XM_004307470*; *FvH4_7g22410*; *gene26612*) as a housekeeping gene. The *Fragaria vesca* eFP Browser (Darwish et al., 2013) provided us with a basis from which we selected FvACT as a housekeeping gene (Hollender et al., 2014), since it shows less variability in expression for all developmental stages studied than other housekeeping genes. The specific primers used are described in Supplementary Table S1 and PCR amplicons were sequenced to confirm specificity.

### Statistical analysis

Data were analyzed by ANOVA (one-way analysis of variance), and Duncan’s multiple range test was used (IBM Corp. SPSS Statistics version 22.0. Armonk, NY, United States). Statistical significance was assessed at the level *p* ≤ 0.05.

## Results

### Strawberries with contrasting cold storage exhibiting different senescence-like responses and oxidative stress markers

**Figure 1:**
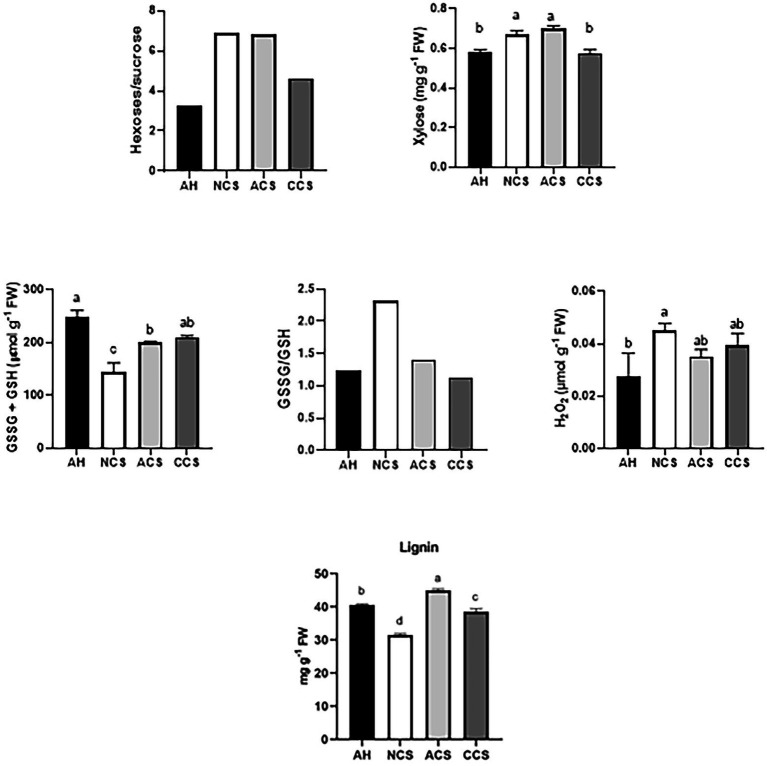
Strawberries with contrasting cold storage conditions exhibiting different senescence-like responses and oxidative stress markers including: ratio of hexoses/sucrose, content of xylose, H_2_O_2_ and glutathione (GSH + GSSG reduced plus oxidized glutathione forms), ratio of GSSG/GSH and lignin content. Strawberries immediately after harvest were used as the control sample (AH). Those placed directly in a chamber at 20°C were used as non-cold stored samples (NCS), while the strawberries transferred at 20°C following cold-stored fruit (ACS) or CO_2_-cold stored fruit (CCS). Graphs represent the average of at least three independent replicates (each one contains 15 fruits). Mean values (*n* = 3 ± standard deviation). (a-c) For each parameter, mean values with different letters are significantly different (*p* < 0.05) according to the Tukey’s multiple range test.

Strawberries immediately after harvest (AH) as a control, those placed directly in a chamber at 20°C for 2 days (NCS) and strawberries transferred at 20°C for 1 day following the 7-day storage at 1°C in air (ACS) or CO_2_-cold stored fruit (CCS) were analyzed. We selected the hexoses/sucrose ratio as indicator of senescence-like process During stress and in senescing leaves, hexose sugars often accumulate, resulting in an increased hexose/sucrose ratio (Wingler and Roitsch, 2008). In addition, we previously reported that CO_2_-treated strawberries reduced sucrose degradation primarily linked to a down-regulation of vacuolar invertase (*FvVINV2*) and cell wall invertase (*FvCWINV1*) transcripts (del Olmo et al., 2020). Although little is known about senescence-specific marker in fruit, we suggest that the relative ratio between hexoses and sucrose rather than the absolute concentration of sugars may be a good marker of senescence-like process in fruit. The content of H_2_O_2_ and glutathione pool was selected to monitor fluctuations in oxidative stress (Figure 1). The content of lignin and the soluble xylose were also determined. The results indicate that the overall ratio of hexoses (glucose and fructose) to sucrose as well as the content of xylose within CCS was the lowest in comparison with ACS and even NCS. The highest content of H_2_O_2_ was found in NCS. Furthermore, NCS samples showed a significant decrease in the glutathione pool and a shift in the GSH redox status, with the glutathione pool becoming more oxidized. The highest amount of lignin was quantified in ACS. These results suggest that strawberries under the three storage conditions are ideal for studying the molecular mechanism of cold storage and the effect of high CO_2_ pretreatment during cold storage to overcome oxidative stress and to repress senescence-like process at consumption.

### Differential expression analysis and gene ontology analysis of strawberries with contrasting cold storage

**Figure 2:**
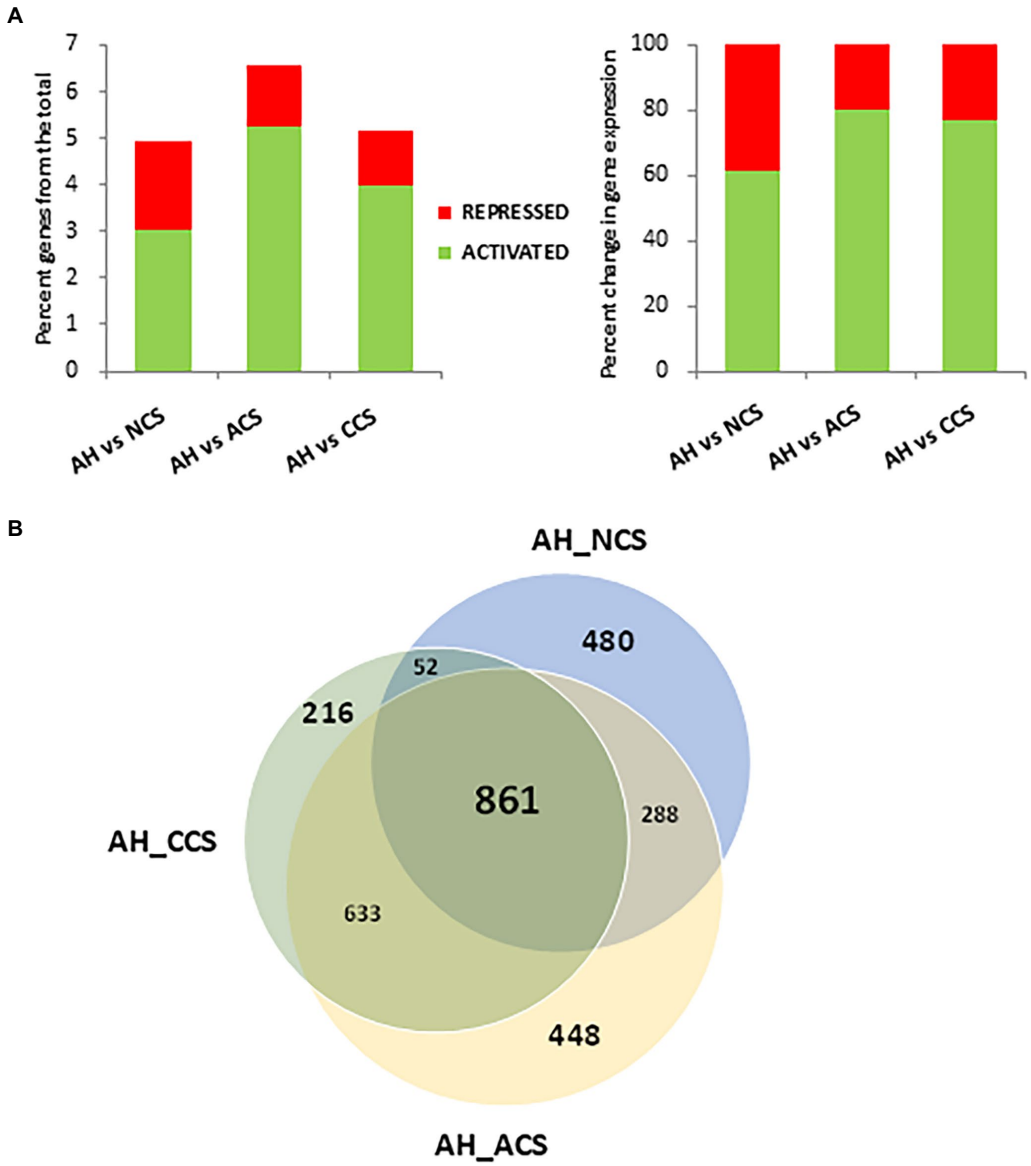
Differentially expressed genes (DEGs) analysis among after harvest (AH) and different cold storage conditions. **(A)** Percentage of differentially activated or repressed genes at global level or among the comparisons: After harvest (AH) vs. non-cold stored (NCS), AH vs. air-cold stored (ACS) and AH vs. CO_2_-cold stored (CCS). **(B)** Venn diagram showing the number of DEGs in the comparisons, AH vs. NCS, AH vs. ACS and AH vs. CCS.

In the differential expression analysis, the gene expression levels of strawberries with contrasting cold storage conditions: non-cold stored (NCS), air-cold stored (ACS), and CO_2_-cold stored (CCS) were compared using a pairwise analysis with fruit at harvest (AH). Out of the total of 34,000 genes annotated in *Fragaria vesca*, 1,681 genes changed their expression in NCS, 2230 in ACS and 1762 in CCS, respectively. Gene expression changes observed at a global level in NCS, ACS, and CCS only represent 4.9, 6.6, and 5.2% of the total strawberry transcriptome (Figure 2A). In CCS, 77.3% (4% of the total) of the genes were activated, with 22.7% (1.2% of the total) being repressed. Venn diagrams summarize the number of overlapping differentially expressed genes in NCS, ACS, and CCS (Figure 2B). The smallest number of differently expressed genes was observed in AH versus CCS.

**Figure 3:**
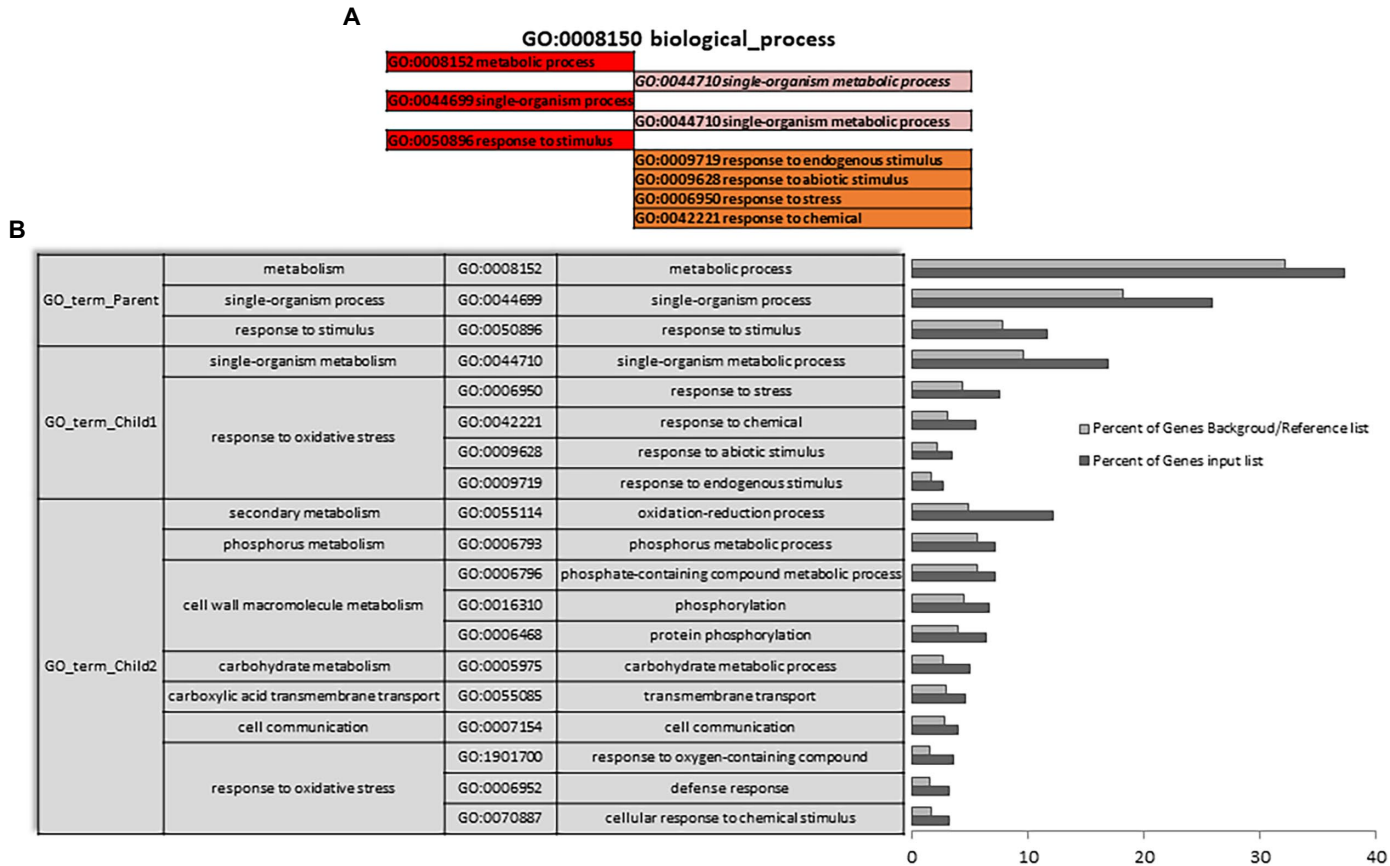
Total gene ontology enrichment. DEGs obtained between after harvest (AH) and non-cold stored (NCS). A Singular Enrichment Analysis (SEA; FDR ≤ 0.05) showing the GO for the most significant biological processes categories obtained comparing AH vs. NCS. **(A)** Scheme of the total enrichment (TE). **(B)** Bar chart of overrepresented terms in biological process category. The *Y*-axis shows the definition of each of the processes included in the first three GO terms annotated. The *X*-axis is the percentage of genes mapped by the term, and represents the abundance of the GO term. The percentage for the input list is calculated by the number of genes mapped to the GO term divided by the number of all genes in the input list. The same calculation was applied to the reference list to generate its percentage.

**Figure 4:**
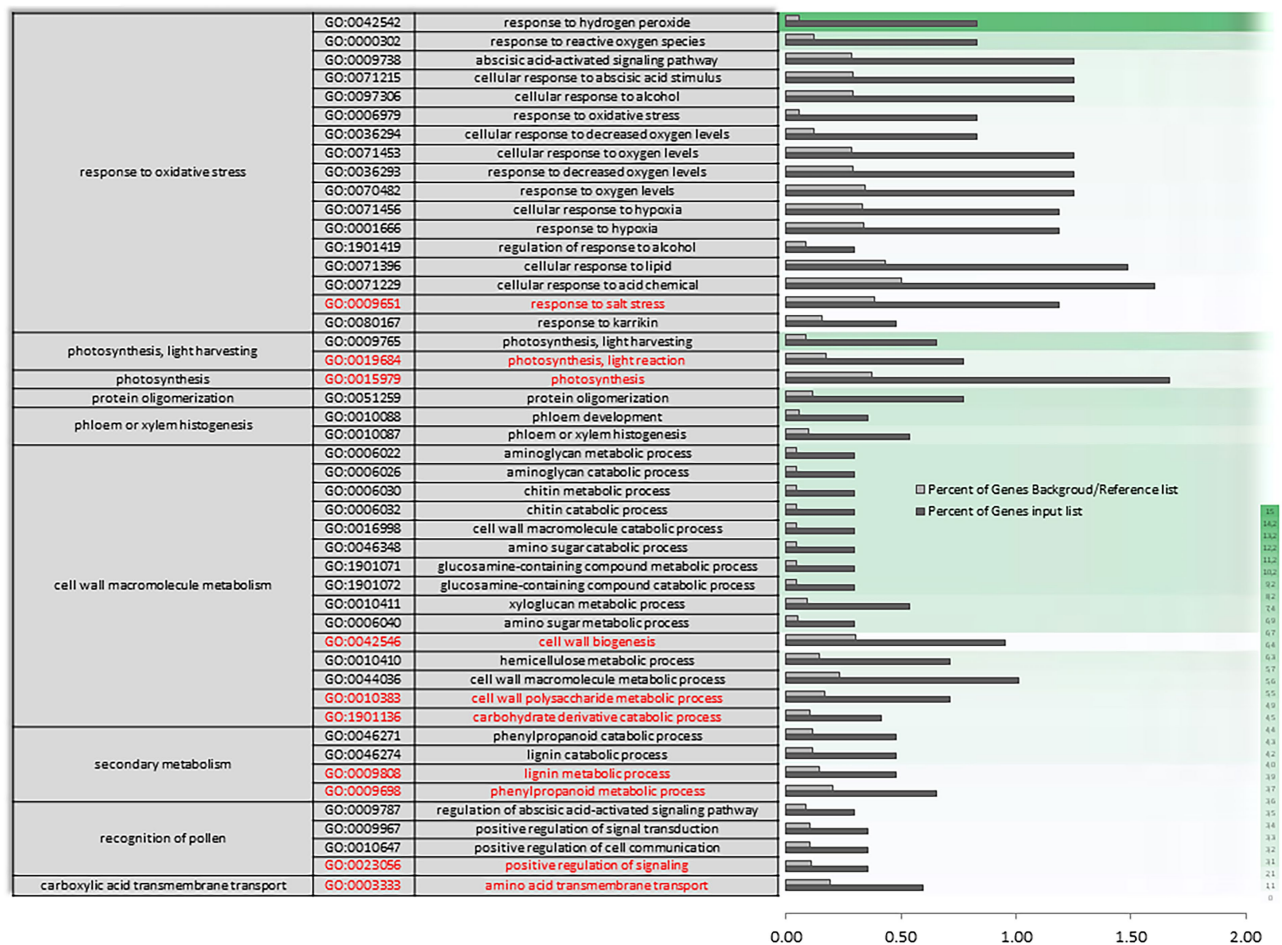
Specific gene ontology enrichment. DEGs obtained between after harvest (AH) and non-cold stored (NCS). Bar chart showing in detail the most significant biological processes terms overrepresented obtaining by a Singular Enrichment Analysis (SEA; FDR ≤ 0.05) in AH vs. NCS comparison. The Y-axis shows the definition of GO terms. The *X*-axis is the percentage of genes mapped by the term, and represents the abundance of the GO term. The percentage for the input list is calculated by the number of genes mapped to the GO term divided by the number of all genes in the input list. The same calculation was applied to the reference list to generate its percentage. These two bars are classificated by a color key showing a green gradient scale for the frequency between both percentages represented.

A Singular Enrichment Analysis (SEA) was performed (FDR ≤ 0.05) for each of the three differential expressions initially analyzed (AH vs. NCS; AH vs. ACS; AH vs. CCS), obtaining a total enrichment (TE) and a specific enrichment (SE) for each of them. Figures 3A,B show how metabolic and response to stimulus processes that were enriched in NCS seem to be closely related to response to oxidative stress processes. As can be seen in Figure 4 there is an enrichment mainly of genes included in the categories with redox and transferase activity. Categories of molecular functions that are unique and specifically enriched (Supplementary Figures S1, S2 in red) include primarily genes encoding proteins with peroxidase activity, transferases that transfer hexosyl groups, together with hydrolases.

**Figure 5:**
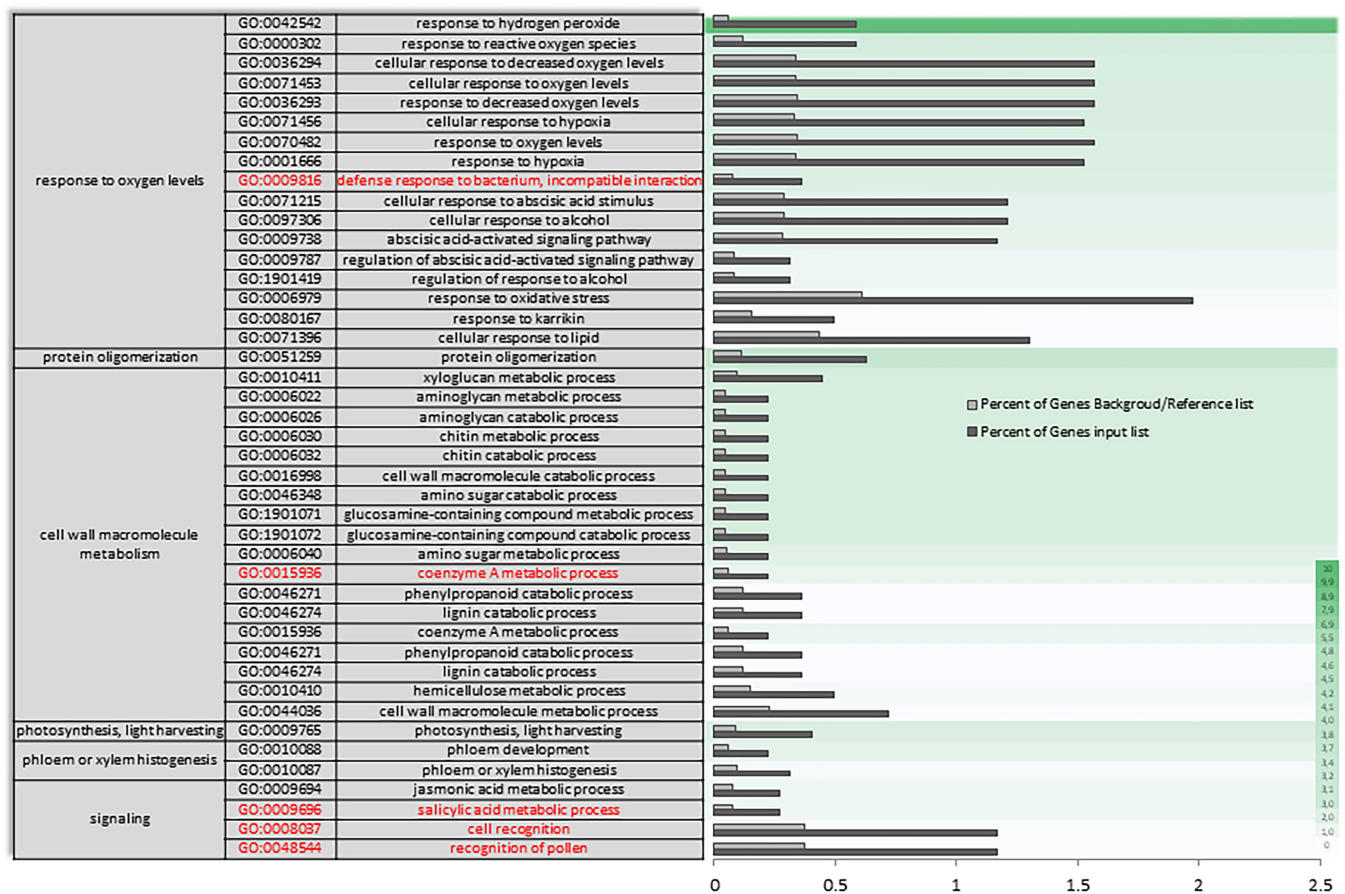
Specific gene ontology enrichment. DEGs obtained between after harvest (AH) and air-cold stored (ACS). Bar chart showing in detail the most significant biological processes terms overrepresented obtaining by a Singular Enrichment Analysis (SEA; FDR ≤ 0.05) in AH vs. ACS comparison. The *Y*-axis shows the definition of GO terms. The *X*-axis is the percentage of genes mapped by the term, and represents the abundance of the GO term. The percentage for the input list is calculated by the number of genes mapped to the GO term divided by the number of all genes in the input list. The same calculation was applied to the reference list to generate its percentage. These two bars are classificated by a color key showing a green gradient scale for the frequency between both percentages represented.

In AH vs. ACS, an increase in the differential expression of genes plays a role in the response to oxygen levels and the multi-organism process (Supplementary Figure S3). The set of differentially expressed oxygen response genes (Figure 5) appears to be mediating in response to hydrogen peroxide and reactive oxygen species, as well as to decreased oxygen levels and hypoxia, and as a cellular response to fermentative alcohol levels (Figure 5 and Supplementary Figure S3 in red). Molecular function categories focus on the regulation of catalytic and transporter activities. Specifically, there is a unique and characteristic differential enrichment (Supplementary Figures S4, S5 in red) in activities such as peroxidase, along with jasmonates.

**Figure 6:**
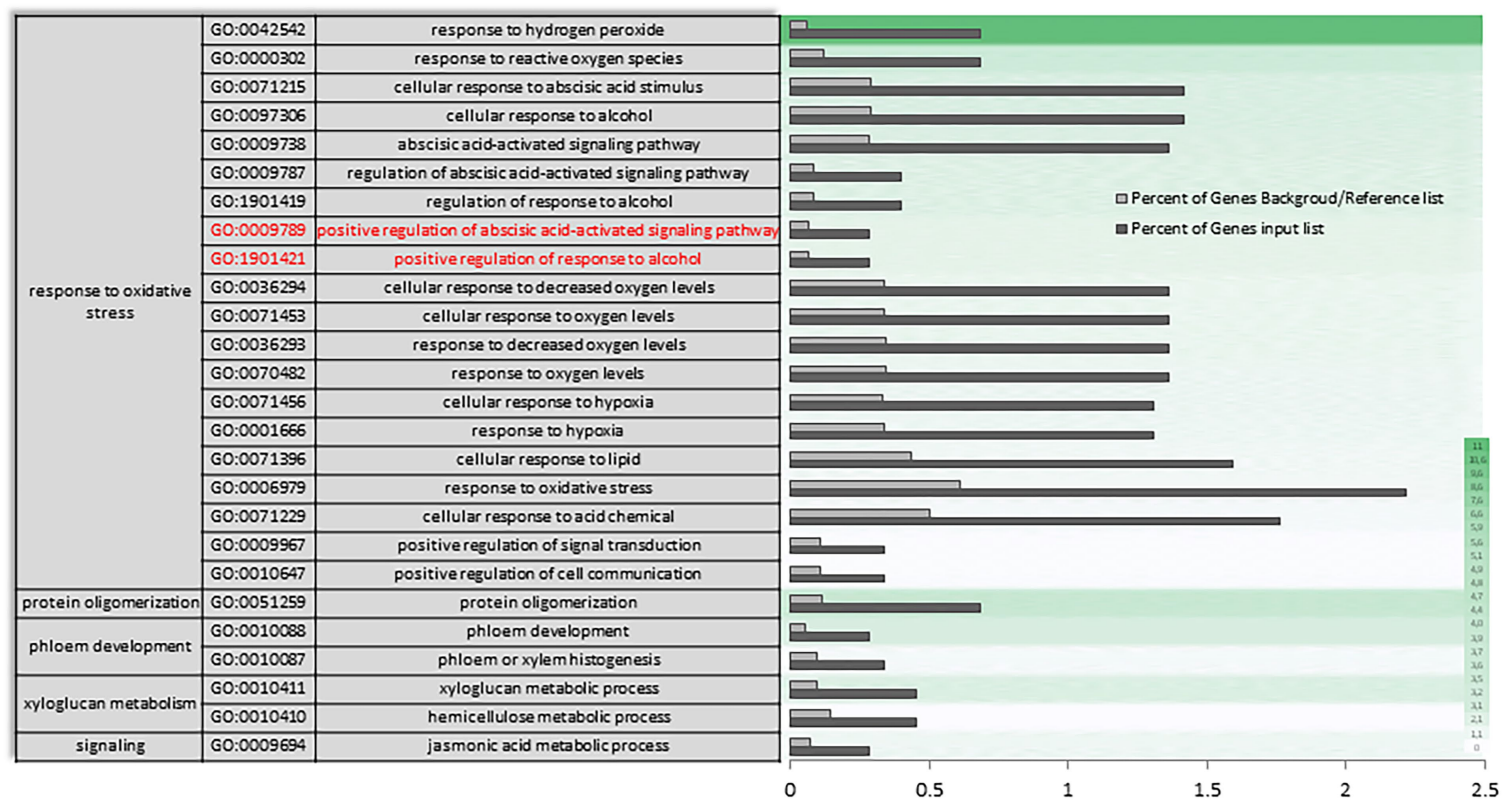
Specific gene ontology enrichment. DEGs obtained between fruit after harvest (AH) and CO_2_-cold stored (CCS). Bar chart showing in detail the most significant biological processes terms overrepresented obtaining by a Singular Enrichment Analysis (SEA; FDR ≤ 0.05) in AH vs. CCS comparison. The *Y*-axis shows the definition of GO terms. The *X*-axis is the percentage of genes mapped by the term, and represents the abundance of the GO term. The percentage for the input list is calculated by the number of genes mapped to the GO term divided by the number of all genes in the input list. The same calculation was applied to the reference list to generate its percentage. These two bars are classificated by a color key showing a green gradient scale for the frequency between both percentages represented.

In the transcriptional reconfiguration that occurs in AH vs. CCS, a single-organism process, stimulus response and signaling have key roles with differential expression of genes involved in xyloglucan (XG) metabolism (Figure 6; Supplementary Figure S6). Specific enrichment also continues to be primarily linked to response to hydrogen peroxide and reactive oxygen species. However, some functional categories enrichment is only observed between both experimental conditions, such as the positive regulation of activation mediated by abscisic acid and in response to alcohol (Figure 6 in red). Regarding the molecular function categories (Supplementary Figure S7), a reorganization occurs in the expressions of some gene categories participating in the control of pectinesterases. A unique and significant differential enrichment can be seen in gene categories of transferase activity, transferring hesoxyl groups (Supplementary Figure S8 in red).

### Genes differentially expressed controlled by high CO_2_

**Figure 7:**
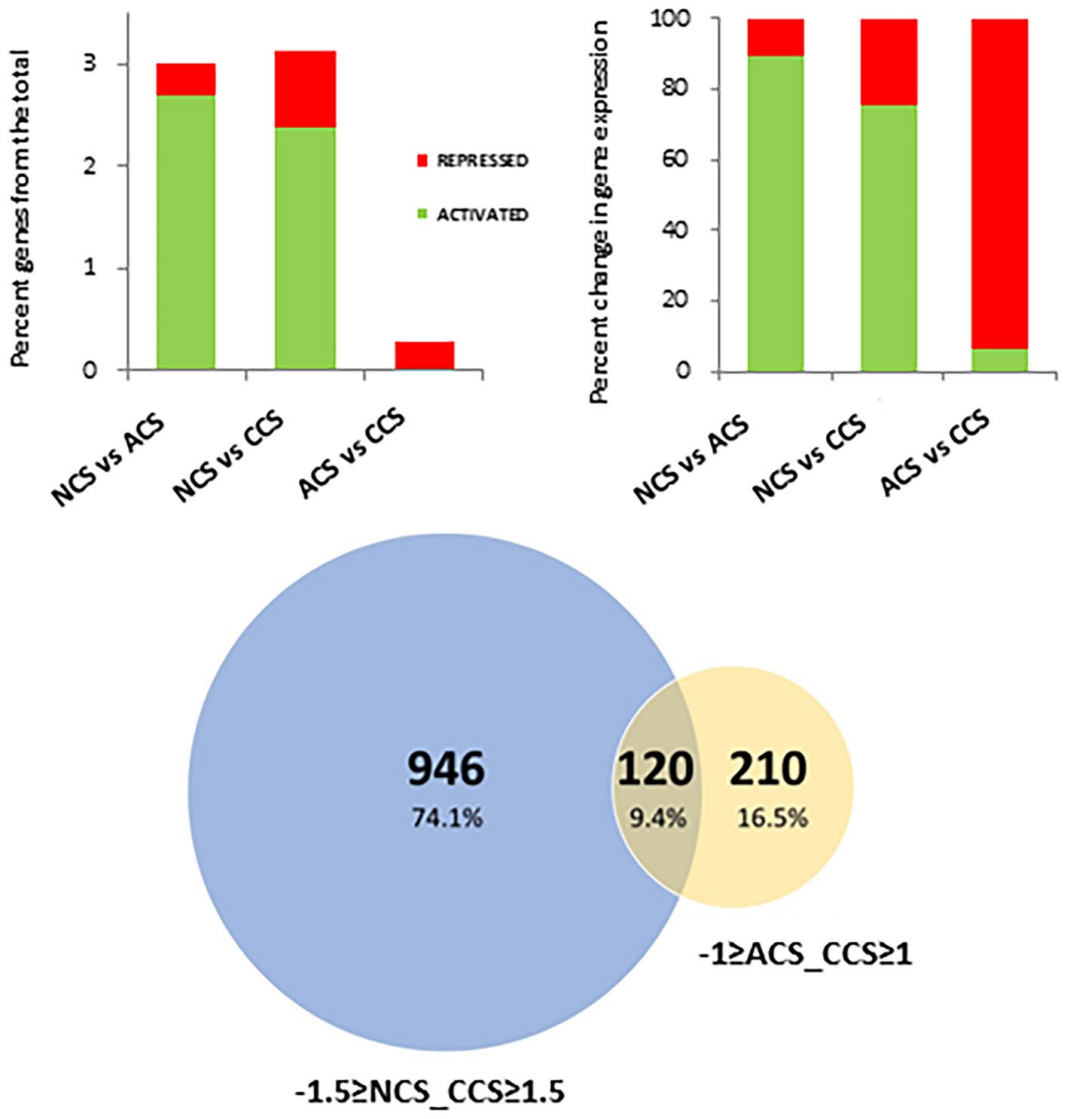
Differentially expressed genes across the different cold storage conditions. (Upper panel) Percentage of differentially activated or repressed genes at global level or among the comparisons: Non-cold stored (NCS) vs. air-cold stored (ACS), NCS vs. CO_2_-cold stored (CCS) and ACS vs. CO_2_-cold stored (CCS). (Lower panel) Venn diagram showing the number of DEGs in the comparisons, −1.5 ≥ NCS vs. CCS ≥ 1.5 and-1 ≥ ACS vs. CCS ≥ 1.

**Figure 8:**
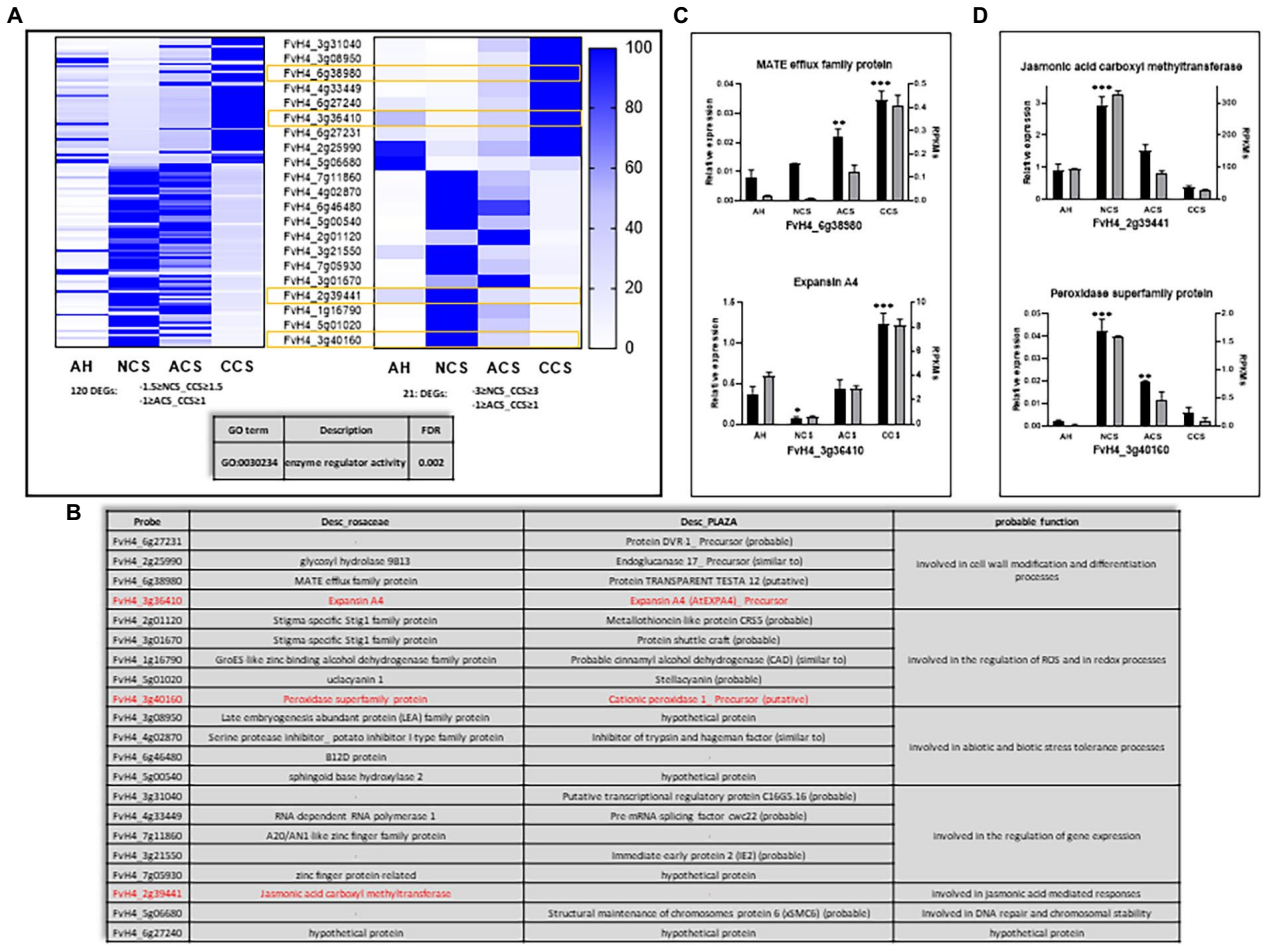
Genes differentially expressed at 20°C controlled by high CO_2_. **(A)** Heatmaps representing Reads Per Kilobase Million (RPKM) expressed as a relative percentage of the DEGs. Left: DEGs obtained from the intersection between-1.5 ≥ NCS_CCS ≥ 1.5 and-1 ≥ ACS_CCS ≥ 1; right: DEGs with highest change obtained from the intersection between-3 ≥ NCS_CCS ≥ 3 and-1 ≥ ACS_CCS ≥ 1, where a yellow square is indicating which genes are analyzed by qPCR. The table at bottom express the biological process term overrepresented obtaining by a Singular Enrichment Analysis (SEA; FDR ≤ 0.05). **(B)** Table showing a representative pool of DEGs regulated by high CO2 with its probable function. Relative expression analysis through qPCR (black bars) vs. RPKMs obtained in RNAseq (gray bars) for the genes: **(C)** FvH4_6g38980 and FvH4_3g36410, that are increasing its expression and **(D)** FvH4_2g39441 and FvH4_3g40160, that are repressing its expression by the CO2 treatment. Graphs represent the average of at least three independent replicates quantified. Error bars indicate ± SD; One-way ANOVA for relative expression of AH versus NCS, ACS, or CCS value. Adjusted *p* value (^*^p ≤ 0.03, ^**^p ≤ 0.002; ^***^p < 0.001).

To identify the genes involved in deteriorative processes controlled by CO_2_, gene expression levels of CCS were compared using a pairwise analysis with NCS and ACS. A total of 1,066 genes were differentially expressed in CCS vs. NCS, corresponding to 3.2% of the total genes. This comprised 2.4% genes activated and 0.8% repressed (Figure 7). The majority of genes, 93.8% (0.3% of the total), are repressed between ACS and CCS, with only 6.2% activated. A total of 120 genes were obtained from the intersection data of NCS and CCS (−1.5 ≥ fold change ≥ 1.5), together with those obtained of ACS and CCS (−1 ≥ fold change ≥1). Figure 8A shows the heatmap of the RPKM of these 120 genes placed by Fc between NCS and CCS, and expressed as a relative percentage, mainly including repressed genes (71/120) and activated ones (43/120) in both DESeq analyses, respectively (NCS vs. CCS and ACS vs. CCS). Also to be noted, a minority of genes are activated between NCS and CCS and repressed between ACS and CCS (6/120). These genes, partially or specifically controlled by CO_2_, were significantly classified exclusively in the molecular function category: enzyme regulator activity (GO 0030234).

But to highlight the genes that undergo the greatest change, we analyzed the 21 genes resulting from the DEseq intersection between NCS and CCS (−3 ≥ fold change ≥3), together with those obtained from DEseq analyses between ACS and CCS (−1 ≥ fold change ≥ 1; Figure 8A, right). Similarly, the majority of genes we obtained were exclusively repressed (12/21) or/and activated only in DESeq analyses (9/21). As can be seen in Figure 8B, we can highlight genes that encode proteins involved in cell wall modification processes, in the regulation of ROS and in redox processes. This analysis also includes different regulators of gene expression, and, to a lesser extent, effectors of JA-mediated responses and factors involved in DNA repair and chromosome stability. We used RT-qPCR to analyze the expression of two genes that are transcriptionally activated (*FvH4_3g36410* and *FvH4_6g38980*) encoding an Expansin A4 and a MATE efflux family protein, respectively (Figure 8C), and two that repress their expression in response to CO_2_ (*FvH4_2g39441* and *FvH4_3g40160*), which encode a JA carboxyl-methyltransferase and a peroxidase superfamily protein, respectively (Figure 8D).

### Effect of high CO_2_ on texture

Different empirical tests have been used to measure texture in fruits and vegetables comprising of living plant cells. However, measured parameters from empirical tests are generally poor measures of perceived texture. In contrast, between imitative methods, Volodkevich bite jaws probe record the force of biting on a piece of food as function of the deformation applied. By using this fixture, it has been shown that the characteristic peaks of the force–distance curve of different fruits and vegetables corresponded well to specific tissue parts and softening-related changes (Alvarez et al., 2020; Kim et al., 2020). In order to analyze the effect of high CO_2_ on texture during cold storage and after transfer to 20°C, mechanical parameters derived from Volodkevich force-distance curves, including first peak force, shearing average force and shearing energy were examined (Table 1). The yield point and the shear energy required to cause an irreversible deformation were the highest at the end of 2 days of high CO_2_ treatment, even 60% more than at harvest time in the case of yield point. Although the force value decreased 5 days after transfer to air, the yield point value was 85% higher than that of air-stored strawberries and similar to fruit at harvest time. After transfer to 20°C, the shearing average force and shearing energy values were higher in CCS than in ACS and similar to fruit at harvest time (AH).

**Table 1:** Mechanical parameters derived from Volodkevich force-distance curves.

Treatments	First force peak (*N*)	Shearing average force (*N*)	Shearing energy (*J*)
**After harvest** (0 day) AH	2.45 ± 0.185^b^	2.73 ± 0.188^a^	0.022 ± 0.002^b,c^
**Kept at 20°C** (2 days) NCS	2.28 ± 0.136^b^	2.17 ± 0.549^b,c^	0.020 ± 0.001^c^
**Storage at 1°C**			
2 days air	1.68 ± 0.107^d^	1.55 ± 0.149^d^	0.014 ± 0.001^d,e^
7 days air	1.93 ± 0.150^c,d^	1.85 ± 0.196^c,d^	0.016 ± 0.001^d^
2 days CO_2_	3.91 ± 0.317^a^	2.79 ± 0.141^a^	0.026 ± 0.001^a^
2 days CO_2_ + 5 days air	2.27 ± 0.145^b,c^	2.87 ± 0.256^a^	0.024 ± 0.002^a,b^
**Transfer to 20°C**			
ACS (air-cold stored)	1.15 ± 0.084^e^	1.44 ± 0.138^d^	0.012 ± 0.001^e^
CCS (CO_2_-cold stored)	1.94 ± 0.076^c,d^	2.49 ± 0.228^a,b^	0.021 ± 0.001^c^

Strawberries were analyzed immediately after harvest (AH). Non-cold stored were placed directly in a chamber at 20°C were analyzed after 2 days (NCS). Cold-stored strawberries in air were analyzed at the early (2 d air) and at the late phase of cold-storage period (7 d air). CO_2_-treated with a continuous flow of a gas mixture containing 18% CO_2_ + 18% O_2_ rest N_2_ were analyzed at the end of CO_2_ treatment (2 d CO_2_) and after five additional days (2 d CO_2_ + 5 d air). Strawberries transferred at 20°C for 1 day following air-cold stored fruit (ACS) or CO_2_-cold stored fruit (CCS). Mean values (*n* = 5 ± standard deviation). ^a–e^For each mechanical parameter, mean values with different letters are significantly different (*p* < 0.05) according to the Tukey’s multiple range test.

### Molecular bases of enhancing firmness by high CO_2_

**Figure 9:**
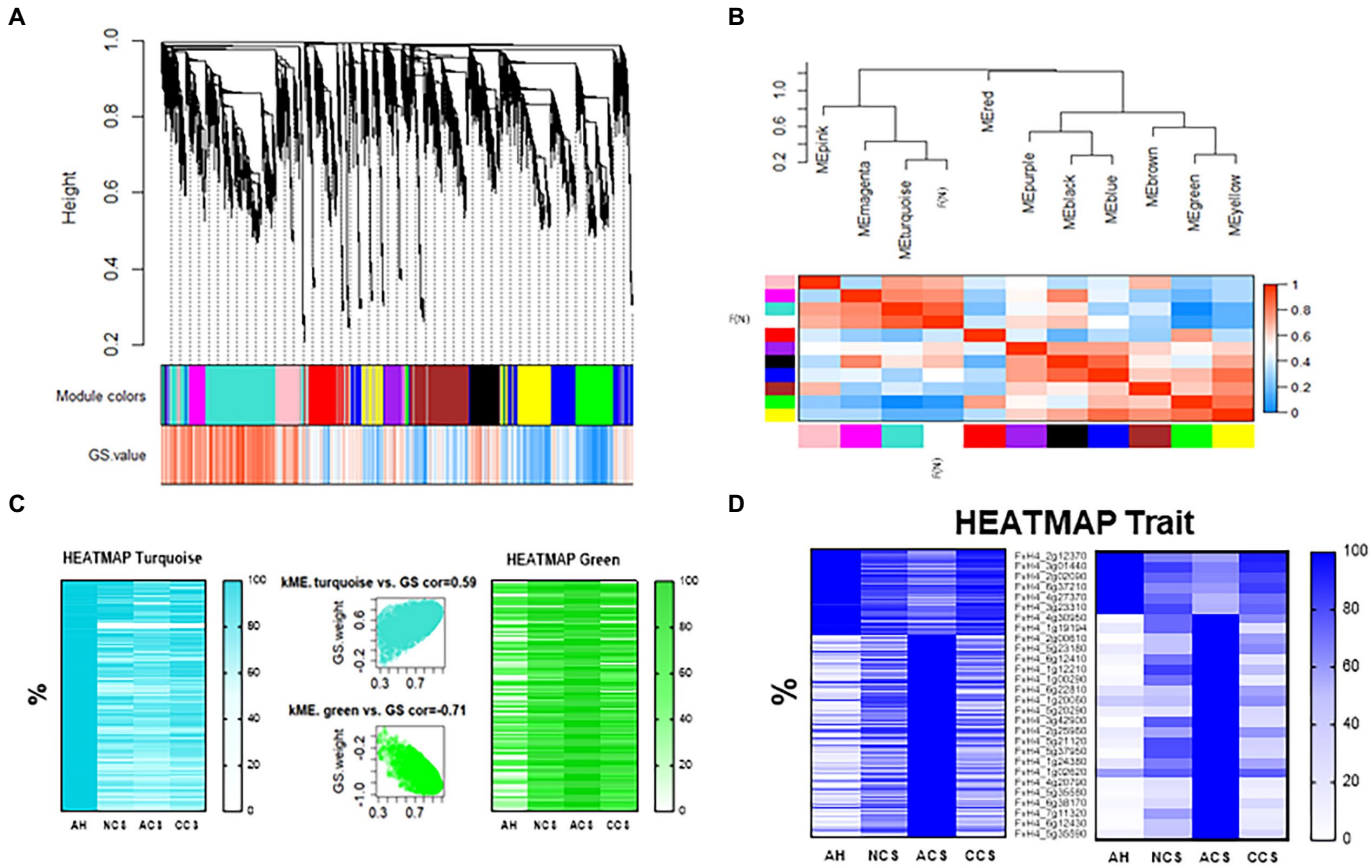
Weighted Gene Correlation Network Analysis. **(A)** Gene cluster dendrogram and module colors assignment for detection of the modules together with the gene significance for body weight (GS. Weight). Colors in the horizontal bar represent the modules. Ten modules with 31,000 transcripts were detected with WGCNA. **(B)** Eigengene dendrogram with an adjacency heatmap for module-trait (shearing average force, F(N)) relationship, the progressively intensity gradient of blue and red colors are indicating a high co-expression interconnectedness. **(C)** Module colors heatmap representing Reads Per Kilobase Million (RPKM) expressed as a relative percentage of genes obtained in turquoise (left) and green (right) modules. In the middle is shown the modules significantly correlated with F(N): module membership (MM) turquoise (middle-up) and green (middle-down) vs. GS. Weight. Each point represented an individual gene within each module, which were plotted by GS. Weight on the *Y*-axis and MM on the *X*-axis. **(D)** Heatmap showing the expression of the 910 genes (left) that mostly correlate with strawberry firmness (−0.85 ≥ kME ≥ 0.85), represented as a relative RPKM percentage of the genes correlated. On right a selection of 28 genes analyzed by qPCR.

Data of shearing average force (*N*; Table 1) was used as a trait in the WGCNA analysis. The initial result of this correlation analysis is shown in Figure 9A, where a hierarchical, weighted cluster tree of the 31,000 genes of *Fragaria vesca* is presented. This tree allows us to link the displayed color bands, providing a simple visual comparison of each module obtained. So, the first band shows the results of the automatic single block analysis (Module colors), and the second color band provides the measure of genetic significance (GS.value). Thus, Figure 9B illustrates the relationship of each module with the trait “firmness” and how this association allows us to relate each cluster with the rest of the clusters identified as well as with the trait, through the mean of the correlation value between each gene of the module. In the present work, ten modules were detected (Figure 9B). These modules, defined as branches of the cluster tree, were selected with a minimum but relatively large size for the division of each branch in such a way that the visualization of the gene network represents the relationships between the modules and the firmness of the fruit. Hence, the dendrogram branches group densely interconnected and highly co-expressed genes. The cutting method (Langfelder et al., 2008) allowed us to select the turquoise, green, and yellow modules as those that are best associated with firmness and that could mainly give biological significance to the network. Finally, the turquoise and green modules were chosen since they were the ones that showed the highest correlation in the intramodular analysis (kME vs. GS cor), of 0.59 and 0.71, respectively (Figure 9C). In this way, we were able to identify which genes have a high significance for weight (GS), as well as a high membership of modules in the turquoise or green modules (MM), finding 5,665 associated genes in the turquoise module (≈ 18.3% from the total genes analyzed using WGCNA and 2,670 genes in the green module (8.6% from the total genes analyzed using WGCNA. On the other hand, the heatmaps obtained from each module (Figure 9C) show how the relative expression of each gene, ranked by weight (KME), changes according to the treatment applied and its correlation degree with the firmness. The set of genes analyzed in the turquoise module was found to be directly related to firmness (less expression: lower firmness), whereas the set of genes of the green module is inversely related. So, the turquoise module contains more genes with high positive correlations with strawberry firmness, while the green module contains more genes with negative correlations (Figure 9C). Likewise, we can observe in Figure 9D that a detailed analysis for the trait “firmness” was obtained together with the weight of each module. Thus, the heatmap in Figure 9D (left) shows the expression of the 910 genes that mostly correlate with strawberry texture (−0.85 ≥ kME ≥ 0.85).

**Figure 10:**
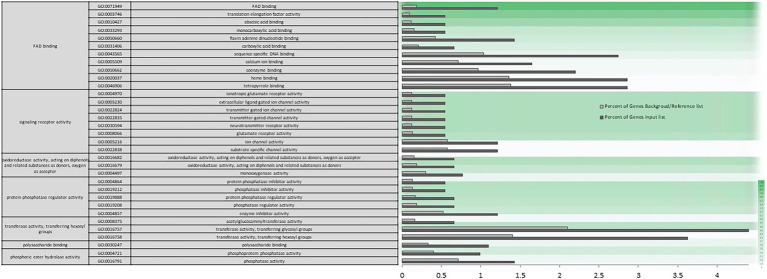
Specific gene ontology enrichment obtained for firmness using Weighted Gene Correlation Network Analysis. Bar chart showing in detail the most significant molecular function terms overrepresented obtaining by a Singular Enrichment Analysis (SEA; FDR ≤ 0.05) in WGCNA. The *Y*-axis shows the definition of GO terms. The *X*-axis is the percentage of genes mapped by the term, and represents the abundance of the GO term. The percentage for the input list is calculated by the number of genes mapped to the GO term divided by the number of all genes in the input list. The same calculation was applied to the reference list to generate its percentage. These two bars are classificated by a color key showing a green gradient scale for the frequency between both percentages represented.

The biological function category enriched in the firmness trait using WGCNA include genes involved in metabolic processes, signaling, and regulation of cellular processes (Supplementary Figures S9, S10). Nevertheless, regarding the biological processes that play a preponderant role in fruit firmness, it is worth highlighting the important specific genes enrichment involved in metabolic processes of jasmonic acid, genes involved in defense response to biotic stimuli and in the immune response regulation, as well as hypoxia response genes. The total GO enrichment of molecular function processed (Supplementary Figure S11) involved in catalytic transferase, oxidation–reduction and isomerase activities take on importance, but also in binding to cofactors, hormones, and carbohydrates, as well as the regulation of the activity of transmembrane transporter and involved in signal transduction. The specific enrichment in gene categories of molecular function focuses on genes involved in FAD binding and redox activity (Figure 10).

**Figure 11:**
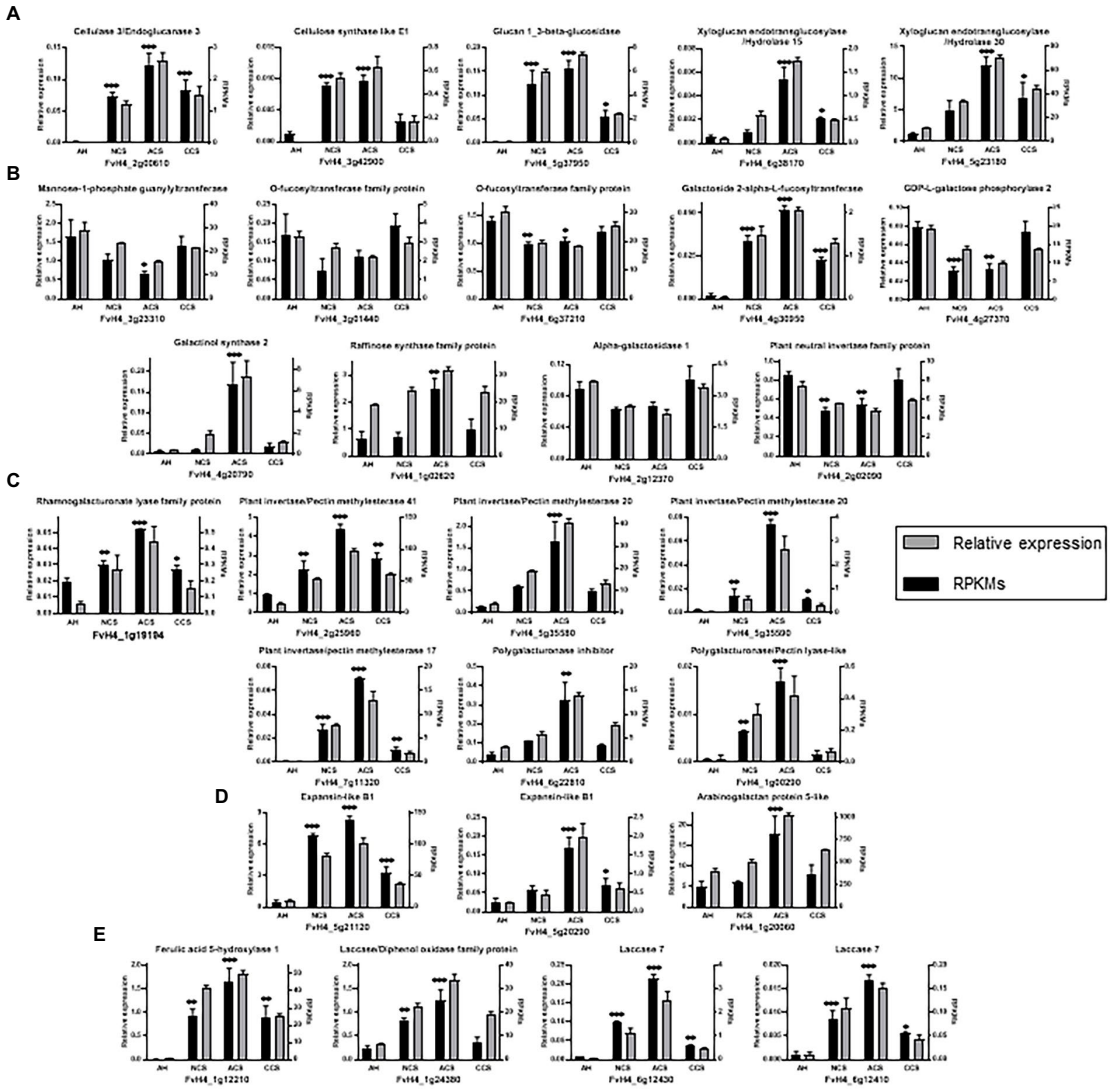
Expression analysis of a group of 28 genes co-expressed by Weighted Gene Correlation Network Analysis. Relative expression analysis through qPCR (black bars) vs. RPKMs obtained in RNAseq (gray bars) for genes involving: **(A)** metabolism of cellulose and hemicellulose, **(B)** N-glycosylation processes and cell wall simple sugars and polysaccharides metabolism, **(C)** pectins metabolism, **(D)** cell expansion and plant growth **(E)** and lignin metabolism. Graphs represent the average of at least three independent replicates quantified. Error bars indicate ± SD; One-way ANOVA for relative expression of AH versus NCS, ACS, or CCS value. Adjusted *p* value (^*^p ≤ 0.03, ^**^p ≤ 0.002; ^***^p < 0.001).

Most of the enrichments described for these 910 genes with maximum correlation with fruit firmness are contemplated in the 28 genes analyzed by qPCR. So, these genes are enriched in biological processes involved in carbohydrate metabolism and cell wall organization. Similarly, these 28 genes analyzed are classified into molecular function categories enriched in hydrolase and transferase catalytic activities (FDR < 0.05). In the qPCR analyses carried out to verify these results, we examined genes involved in the organization of the different components of the cell wall, including cellulose and hemicellulose (Figure 11A), activated sugars, simple sugars and oligosaccharides (Figure 11B), pectins (Figure 11C), proteins (Figure 11D), and lignin (Figure 11E).

**Figure 12:**
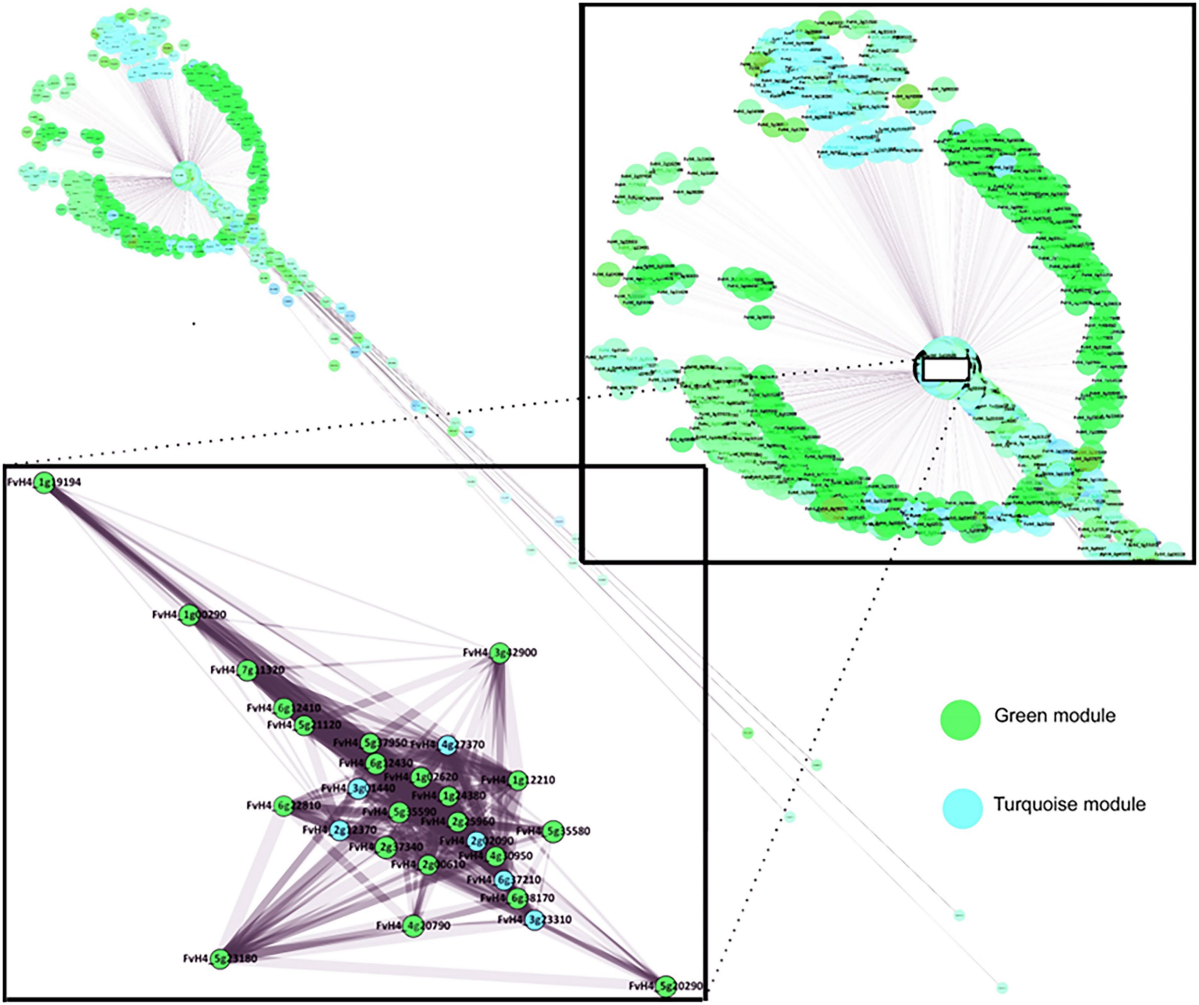
Weighted gene co-expression network for firmness of Mara des Bois strawberries at 20°C controlled by high CO_2_. Network for the genes co-expressed in turquoise and green modules which have a high weight over firmness. The inverse relationship clusters connecting fruit firmness with biotic stress and involving cell wall homeostasis are occupying a central place in the gene expression network. The close-up is highlighted a representative group of genes for both modules analyzed by qPCR.

An analysis was also performed of the location of genes in the co-expression network generated through WGCNA (Figure 12). This network gives more biological significance to this set of genes since it is built from all the gene modules that characterize the studied correlation. As can be seen in Figure 12, these selected genes cluster in a small area, suggesting that their expressions could be mutually dependent or co-regulated. In addition, this grouping of genes seems to be highly focused on the global set of the co-expression network, which suggests that they could be part of the basic mechanisms that control fruit softening.

## Discussion

### The differential expression analysis showed an enrichment in genes involved in oxidative stress in non-cold stored and in response to abiotic stress in air-cold stored strawberries

Through exploring sets of DEGs for higher-level biological molecular themes based on GO and pathway assignments as well as the changes in the quantitative measures of oxidative stress responses, our results indicate that the metabolic process occurring preferentially in non-cold stored (NCS) fruit was closely related to response to oxidative stress. The categories that undergo the greatest change in expression in NCS samples focus on the response to hydrogen peroxide and cell wall macromolecular metabolism (see AH vs. NCS, Figures 3, 4). Furthermore, the highest values of H_2_O_2_ and the decrease in GSH (Figure 1) support the view that NCS samples have a reduced capacity for scavenging ROS. Kumar and Chattopadhyay (2018) reported that GSH plays a role in inducing heat shock proteins, which is vital for combating various stresses, including oxidative stress (Wang et al., 2014).

In the air-cold stored (ACS) samples, the greatest variation observed in the functional categories is related to the positive regulation in response to abiotic stress (Figure 5). Plants exposed to stressful cold storage react with a broad range of defense responses in an attempt to prevent damage (Lapous et al., 1998). Our results indicate an increase in the differential expression of genes involved in response to oxygen levels, and especially in response to hydrogen peroxide. Xiao et al. (2018) reported that as ripening progressed, the minimum internal oxygen concentration approached zero correlated with the profile of cell death. If the availability of oxygen to ACS samples is limited by the specific characteristic of epidermal and hypodermal external tissues, fermentative metabolites may be produced even under aerobic conditions. A decrease in cytosolic pH is an early cellular response that typically accompanies anoxia (Gout et al., 2001) and chilling damage (Muñoz et al., 2001). Correlation between cytoplasmic acidification and lignin production in different plant cell suspensions has been reported (Hagendoorn et al., 1994). Lignin production by cell wall damage has also been found (Denness et al., 2011). The highest amount of lignin was quantified in ACS samples (Figure 1).

In CO_2_-cold stored (CCS), the overall ratio of hexoses to sucrose was the lowest in comparison with ACS and even NCS, indicating senescence attenuation. An increase in this ratio in senescing leaf cells has been reported (Wingler et al., 2009). Interestingly, there was a less intense change in the transcriptome with a lower number of genes differentially expressed in CCS samples. We previously reported a lower number of genes with changed expression in response to high CO_2_ at the end of treatment than in air-stored table grapes (Rosales et al., 2016). The DEGs observed in AH vs. CCS conditions (Figure 6 and Supplementary Figures S7, S8) show enrichment in gene categories involved in carbohydrate and XGs metabolic processes. The lower values of soluble xylose in CCS (Figure 1) supports the relevance of XGs by high CO_2_.

### Genes differentially controlled by high CO_2_ treatment associated with the maintenance of cell turgor and the resistance to senescence

When examining the 12 individual genes differentially controlled by high CO_2_ which exhibited the strongest altered response (Figure 8C), the up-regulation of *FvH4_6g38980*, encoding the MATE efflux family protein in CO_2_-treated fruit could be part of the molecular mechanism mediating the accumulation of osmolytes which is essential for maintaining the cell turgor. The plant MATE transporters are important for the accumulation of a broad range of metabolites in organelles and do not require high-energy metabolites (Kusakizako et al., 2020). Therefore, here, we suggest that up-regulation of MATE transports in CO_2_-treated fruit could be part of the molecular mechanism mediating the accumulation of osmolytes which are essential for maintaining the cell turgor concomitant with an efflux of H^+^ ions that can contribute to pH homeostasis (pH regulation). *EXPA4* was also transcriptionally up-regulated by high CO_2_ levels. In the case of *EXPA4* (Harrison et al., 2001) indicated that its expression was very different from the other expansins, suggesting that this expansin appears to act independently of the cell wall breakdown. Meanwhile, the opposite trend in the expression of *EXPA4* between NCS and CCS samples and the down-regulation in NCS samples compared to fruit at harvest may indicate a possible role in the control of senescence. Expansins are associated with enhanced tolerance to oxidative stress (Han et al., 2015). In agreement with this idea, it is likely that the marked transcript abundance for *EXPA4* in CO_2_-treated fruit becomes more tolerant to oxidative stress after harvest. Another gene activated by high CO_2_, *FvH4_3g08950*, encodes for late embryogenesis abundant proteins (LEA). LEA proteins are involved in the general protecting response to many adverse conditions, mainly drought (Jia et al., 2020). In CO_2_-treated table grapes, the accumulation of the LEA protein Group 2 (also known as dehydrins) seems to play a more effective role in maintaining the structure and helping to reduce water loss and senescence-related disorders (Vazquez-Hernandez et al., 2018). All these results indicate that the ability to maintain cell water status through osmotic adjustment for controlling cellular turgor is a clear advantage of high CO_2_-treated strawberries. Additionally, in CCS the specific enrichment in gene categories of molecular function focused on genes involved in flavin-adenine dinucleotide (FAD)-binding proteins. One of the most thoroughly studied FAD-containing families is represented by the enzyme glutathione reductase (GR). FAD is necessary for GR enzyme to convert oxidized glutathione (GSSG) to the reduced glutathione (GSH). We previously reported the beneficial effect of high CO_2_ levels during cold storage was loosely connected with GSH regeneration in strawberries by GR activity (Blanch et al., 2013).

Two genes that showed a marked down-regulation by high CO_2_ encode a JA carboxyl-methyltransferase (JMT) and a peroxidase superfamily protein (POX), respectively (Figure 8D). The down-regulation of *POX1* and *JMT* in CCS follows the results of H_2_O_2_ and lignin, respectively (Figure 1). By contrast, a marked increase in the expression of PER in NCS fruit follows the results of H_2_O_2_ (Figure 1), showing that the response to hydrogen peroxide was the biological process markedly induced in non-cold stored fruit kept at 20°C after harvest. It has been reported that as the fruit ripens, oxidative stress progressively augments (Decros et al., 2019), and ROS accumulation peaks once at the start of ripening and again at overripening. ROS also affects the production of jasmonates (Shumbe et al., 2016). CO_2_-treated strawberries showed the lowest transcript levels of genes involved in jasmonic biosynthetic genes including (*JMT*, *FvH4_2g39441*), (*OPDA-reductase 3*, *FvH4_1g08453* and *FvH4_1g08454*), (*OPDA-reductase 2*, *FvH4_5g32680*) and (*AOS*, *FvH4_5g16260* and *FvH4_2g07421*). Jasmonates are important cellular regulators mediating the senescence process (Thakur et al., 2016), promoting leaf senescence at the transcriptional level by activating a subset of SAGs (senescence-associated genes). In this respect, an up-regulation of *FvH4_3g06513* and *FvH4_1g25983* was observed in NCS and ACS, while a decrease was found in CCS samples. These gene expression changes are consistent with the selected senescence indicator (Figure 1). The significant down-regulation of JA-related genes in CCS samples suggests a possible involvement of decreased levels of JA in senescence-like process attenuation in CO_2_-treated strawberries.

### Molecular determinants of firmness enhancement and cell wall integrity in high CO_2_-treated strawberries

During cold storage and interestingly after transfer to 20°C (Table 1), the shearing average force and the shearing energy values were higher in CCS than in ACS and even higher than NCS. The molecular effect of high CO_2_ on delayed strawberry firmness during cold storage has been analyzed using a heterologous cDNA microarray (Ponce-Valadez et al., 2009) or transcriptomic analysis (Bang et al., 2019). However, it is the first time that the key genes related to resistance to softening and cell wall disassembly in CO_2_-treated following transference from cold storage at 20°C have been identified. We performed WGCNA of the comparative transcriptomes to build networks and define highly interconnected genes involved in the organization of the different components of the cell wall (Figure 9).

With respect to cellulose (Figure 11A), our data indicate that while the gene (*FvH4_2g00610*, *EG3*), is practically absent in fruit at harvest, it is significantly up-regulated in all storage conditions and mainly in ACC. According to Mercado et al. (2010) *FaEG3* does not play a key role in fruit softening in transgenic strawberry plants. However, its silencing affects the amount and, albeit in a minor way, the size of hemicellulosic polymers. The expression of the gene (*FvH4_3g42900*, *CslE1*), was markedly repressed in CCS resembling that of fruit at harvest. In rice, *CslE1* has been reported as a drought response gene (Jung et al., 2021). Considering the significantly lower expression levels of *CslE1* gene in CCS, showing lower water loss, we suggest a possible expression of *CslE1* in response to water loss after harvest.

As mentioned above, the result derived from the WGCNA confirms that XG reorganization plays a crucial role in high CO_2_-treated strawberries. The gen (*FvH4_5g37950*, *β-glc*) which was markedly repressed in CCS resembling that of fruit at harvest, suggest a limited degradation of 1,3-β-glucans in CO_2_-treated strawberries and a lower XG disassembling (Minic, 2008) and a lower liberation of α-glucose form the non-reducing end of β-1,3-glucan. Transcription of *β-glc* increased in response to abiotic stress such as drought and low temperature (Romero et al., 2008; Houston et al., 2016). Also, *XTH30* (*FvH4_5g23180*) and *XTH15* (*FvH4_6g38170*) were significantly down-regulated in CCS samples which showed the highest firmness values. These results follow the changes in xylose that ascertains the possibility of a lower solubilization and hydrolysis of xyloglucan oligosaccharides in the cell wall. From the published transcriptome data set of all *FvXTHs*, (Witasari et al., 2019) reported that the higher expression of *FvXTH9* and *FvXTH6* accelerated strawberry fruit ripening. However, there are few reports on environmental factors regulation of *XTHs* expression. Thus, the lower expression of *β-glc* and *XTH* in CO_2_-treated fruit and the steady state of xylose content, can be linked with maintaining the potentially important functions of XG in the cell wall remodeling and flexible accommodation to conserve turgor pressure.

Notably, in the selected genes cluster (Figure 12), the expression of genes encoding glycosyltransferases (GTs), mainly nucleotide-diphospho-sugar transferase (NDT) family protein that catalyze the transfer of nucleotide sugars are weakly associated with many other genes involved in cell wall metabolism. In the case of GDP-mannose, our results indicate down-regulation of the gene (*mannose-1-phosphate guanylyltransferase* (*GMP*), *FvH4_3g23310*) in ACS (Figure 11B). The *Arabidopsis* mutant *cyt1* deficient in GMP showed severe phenotypes, including deficiencies in cell wall (Lukowitz et al., 2001). GDP-mannose is one of the sources of GDP-β-L-fucose, and its addition carried out by fucosyltransferases (FUTs; Park and Cosgrove, 2015). Our results demonstrate that the expression of *FUT* genes (*FvH4_3g01440*, *FvH4_6g37210*) and (*GGalPP*; *FvH4_4g27370*) did not decrease in CCS. Therefore, we propose that having a similar CO_2_-treated fruit expression of *GMP*, *FUT* and *GGalPP* to freshly harvested ones could be essential for preserving the polymerization of XGs. GDP-mannose and GDP-galactose are also involved in the first steps of the ascorbic acid biosynthesis (Badejo et al., 2008). The metabolism of activated sugars is closely related to sucrose catabolism, which we already reported as being controlled by high CO_2_ treatment (del Olmo et al., 2020). UDP-glucose can be converted to other activated sugars and it is also an entrance step to the synthesis of RFOs. The most representative genes that participate in RFOs metabolism are indicated in Figure 11B. Biosynthesis of RFOs begins with the conversion of uridine diphosphate-galactose (UDP-Gal) and myo-inositol to galactinol catalyzed by galactinol synthase (GolS). Raffinose is synthesized by raffinose synthase through the transfer of a galactosyl moiety from galactinol to sucrose. Galactose can be removed from RFOs through the action of α-galactosidase. The present findings in air-cold stored (ACS) samples show an up-regulation of *FvH4_4g20790* and *FvH4_1g02620*, which are encoding a galactinol synthase 2 (GolS2) and a raffinose synthase (RafS) family protein, respectively. Also, there is a decrease in the expression of *FvH4_2g12370*, encoding an α-galactosidase 1. The present results follow the quantitative determination of raffinose family of oligosaccharides (RFOs; raffinose and stachyose) in strawberries (del Olmo et al., 2020), indicating a sustained synthesis of RFOs in stressed air-cold stored strawberries. Accumulation of trisaccharide raffinose has been also described as a cold-inducible biosynthetic route in other fruit and vegetables (Bustamante et al., 2016; Blanch et al., 2017) and different functional roles have been suggested for RFOs (Elsayed et al., 2014). Considering the selected genes cluster our results evidence that RFOs are responsive responses to cold-induced cell wall disassembly. By contrast, CO_2_-treated strawberries are able to maintain cell wall integrity and up-regulation of RFOs biosynthesis does not happen.

CO_2_-treated fruit showed a down-regulation of genes encoding rhamnogalacturonate lyase (*RG-lyase*), and pectin esterase (*PME/PMEI*; Figure 11C). The gene (*FvH4_1g00290*, *PL*) was considerably repressed in CCS, resembling data found in fruit at harvest, while they increased in ACS, which underwent rapid softening. In transgenic strawberries, suppression of *PL* resulted in firmer fruit (Santiago-Doménech et al., 2008). The marked low expression levels of *RG-lyase*, *PME* and *PL-like* genes in CCS could avoid pectin degradation and agree with the well-known reported presence of a pectin-rich middle lamella by effect of high CO_2_ treatment.

The lowest expression of *EXP-like B1* (encoded by *FvH4_5g21120*) in CCS (Figure 11D) could be associated with a possible reduced modification of xyloglucans in response to high CO_2_. Expansins seem to contribute to cell wall disassembly, weakening the hydrogen bonds between cellulose microfibrils and xyloglucan, and thus increasing the accessibility of wall polymers to hydrolytic enzymes and the incorporation of new polymers into the expanding cell wall. By contrast, as we mentioned above, in the case of *EXPA4*, the highest level of expression was found in CCS samples. The marked increase in the expression of *EXPA4* remains an active area of research. Several studies have also provided evidence that expansins are associated with enhanced tolerance to abiotic stress and influence the activity of cell wall-bound peroxidase (Han et al., 2015), improving the tolerance of transgenic tobacco plants to oxidative stress. In agreement with this idea, it is likely that the marked transcript abundance for *EXPA4* in CO_2_-treated fruit becomes more tolerant to oxidative stress and deterioration after harvest, while the lowest values were found in NCS results in enhanced oxidative stress (amount of H_2_O_2_).

H_2_O_2_ and JA-based signaling processes have been suggested in the regulation of lignin production by cell wall damage (Denness et al., 2011). Our results indicate that lignin accumulation is particularly relevant in ACS (Figure 1), even though they become completely softened. Lignin in ACS was linked to a markedly enhanced expression of *CAD* (*FvH4_1g16790*), *F5H* (*FvH4_1g12210*), as well as a set of laccases (*FvH4_1g24380*, *FvH4_6g12430*, and *FvH4_6g12410*; Figure 11E). At the last step of lignin, CAD enzyme converts three types of hydroxycinnamoyl aldehydes into their corresponding hydroxycinnamoyl alcohols (monolignols). The cell wall stiffening by lignification is considered a terminal process in specific cells and deposition of lignin in response to different kinds of stresses and cell wall damage (Denness et al., 2011; Le Gall et al., 2015). Hence, we suggest that lignification in ACS may be a compensatory protective barrier in response to cell wall polymers degradation caused by stress during cold storage in air. This cold stress has been overcome in CCS by pretreatments with high CO_2_ levels maintaining cell wall integrity, and so no lignification has happened.

## Conclusion

We performed a global transcriptome analysis in *Fragaria vesca* using RNA-seq for an integrative study of texture at consumption, an obvious target for preventing fruit loss and to gain quality, as well as to identify the molecular determinants underlying the protection triggered by high CO_2_ to avoid deteriorative processes. Through exploring sets of DEGs for higher-level biological and molecular themes based on GO and pathway assignments, strawberries kept at 20°C after harvest are characterized by an acute enrichment in genes mainly involved in oxidative stress and up-expression of genes involved in jasmonate biosynthesis that mediate the senescence process in strawberries after harvest. WGCNA of the comparative transcriptome identified key genes which might be associated with cell wall elasticity of firmer CO_2_-treated strawberries. The decrease in the expression of *Csl E1*, *XTH30*, *EXP-like E1* and the maintenance of initial steady-state expression levels of genes involved in transfer of activated sugars such as *GMP* and *FUT* may be the cause of the beneficial effect of high CO_2_ primarily maintaining the crosslinks as well as stabilizing xyloglucans. The genes involved in rhamnogalacturonan I and homogalacturonan, mainly *RG-lyase* and *PL-like* are considerably down-regulated in firmer CO_2_-treated fruit, resembling the expression of fruit at harvest and may explain the improved middle lamella integrity. Furthermore, the up-expression of *EXPA4*, *LEA*, and *MATE* genes in CO_2_ treated strawberries, indicating their ability to preserve turgor pressure through sucrose and solutes accumulation and transport into the vacuole. By contrast in soft stressed air-cold stored samples there is a decrease in XG cross-linking along with highly disrupted pectins. Furthermore, the transcriptome data underline the preferential transcript accumulation of genes involved in lignin synthesis and raffinose pathway that could be consider as responsive responses to cold-induced cell wall disassembly. The present results on transcriptomic analysis of CO_2_-treated strawberries with enhanced resistance to softening and oxidative stress at consumption will help to improve breeding strategies of both wild and cultivated strawberries.

## Data availability statement

The original contributions presented in the study are publicly available. This data can be found at: https://www.ncbi.nlm.nih.gov/geo/query/acc.cgi?acc=GSE207254.

## Author contributions

CM conceived and designed the experimental setup. IO, IR, and MS-B designed the RNA analysis. RT conducted technical support. MA conducted the texture analyses. ME conducted biochemical analysis. IO carried out the transcriptome analyses. IO and CM analyzed the results and wrote the manuscript. IR assisted in writing the manuscript. All authors contributed to the article and approved the submitted version.

## Funding

This research was supported by the Spanish National R&D&I Plan of the Ministry of Science and Innovation [grants AGL2017-85291-R (ERDF) and PID2020-113965RB-I00/ AEI/10.13039/501100011033].

## Conflict of interest

The authors declare that the research was conducted in the absence of any commercial or financial relationships that could be construed as a potential conflict of interest.

## Publisher’s note

All claims expressed in this article are solely those of the authors and do not necessarily represent those of their affiliated organizations, or those of the publisher, the editors and the reviewers. Any product that may be evaluated in this article, or claim that may be made by its manufacturer, is not guaranteed or endorsed by the publisher.

## Footnotes

^1^ http://www.bioinformatics.babraham.ac.uk/projects/fastqc/

^2^ http://omics.pnl.gov/software/venn-diagram-plotter

## Supplementary material

SUPPLEMENTARY FIGURE S1 Total gene ontology enrichment. DEGs obtained between AH and NCS. Bar chart showing in detail the most significant molecular function terms overrepresented obtaining by a Singular Enrichment Analysis (SEA; FDR ≤ 0.05).

SUPPLEMENTARY FIGURE S2 Specific gene ontology enrichment. DEGs obtained between AH and NCS.

SUPPLEMENTARY FIGURE S3 Total gene ontology enrichment. DEGs obtained between AH and ACS. Total gene ontology enrichment. A Singular Enrichment Analysis (SEA; FDR ≤ 0.05) showing the GO for the most significant biological processes categories.

SUPPLEMENTARY FIGURE S4 Total gene ontology enrichment. DEGs obtained between AH and ACS. Bar chart showing in detail the most significant molecular function terms overrepresented obtaining by a Singular Enrichment Analysis (SEA; FDR ≤ 0.05).

SUPPLEMENTARY FIGURE S5 Specific gene ontology enrichment. DEGs obtained between AH and ACS.

SUPPLEMENTARY FIGURE S6 Total gene ontology enrichment. DEGs obtained between AH and CCS. A Singular Enrichment Analysis (SEA; FDR ≤ 0.05) showing the GO for the most significant biological processes categories.

SUPPLEMENTARY FIGURE S7 Total gene ontology enrichment. DEGs obtained between AH and CCS. Bar chart showing in detail the most significant molecular function terms overrepresented obtaining by a Singular Enrichment Analysis (SEA; FDR ≤ 0.05).

SUPPLEMENTARY FIGURE S8 Specific gene ontology enrichment. DEGs obtained between AH and CCS.

SUPPLEMENTARY FIGURE S9 Total gene ontology enrichment obtained for firmness using Weighted Gene Correlation Network Analysis. Bar chart showing in detail the most significant biological processes categories obtaining by a Singular Enrichment Analysis (SEA; FDR ≤ 0.05) in WGCNA.

SUPPLEMENTARY FIGURE S10 Specific gene ontology enrichment obtained for firmness using Weighted Gene Correlation Network Analysis.

SUPPLEMENTARY FIGURE S11 Total gene ontology enrichment obtained for firmness using Weighted Gene Correlation Network Analysis. Bar chart showing in detail the most significant molecular function terms obtaining by a Singular Enrichment Analysis (SEA; FDR ≤ 0.05) in WGCNA.

## Abbreviations

GDR: Genome database for rosaceae
H_2_O_2_: Hydrogen peroxide
JA: Jasmonic acid
RFOs: Raffinose family oligosaccharides
RNA-seq: RNA sequencing
RPKM: Reads per kilobase Million
SEA: Singular enrichment analysis
TEG: Triethylene glycol
TE: Trolox equivalents
WGCNA: Weighted gene co-expression network analysis
