# Supplementary figures and images for: Transcriptomic analysis of CO2-treated strawberries (Fragaria vesca) with enhanced resistance to softening and oxidative stress at consumption
Source: Front Plant Sci. 2022 Aug 19;13:983976. doi: 10.3389/fpls.2022.983976 (PMC9437593; doi:10.3389/fpls.2022.983976)

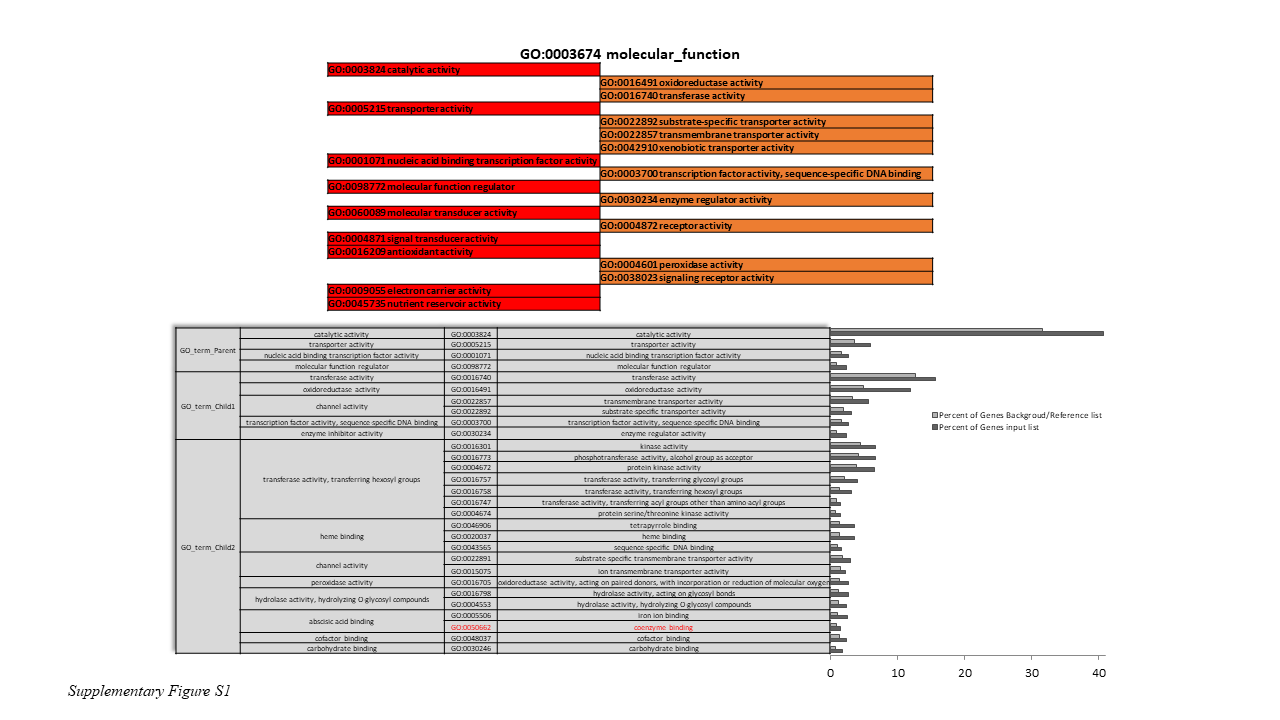

Supplement: SUPPLEMENTARY FIGURE S1 — Total gene ontology enrichment. DEGs obtained between AH and NCS. Bar chart showing in detail the most significant molecular function terms overrepresented obtaining by a Singular Enrichment Analysis (SEA; FDR ≤ 0.05). [file Image_1.tif]

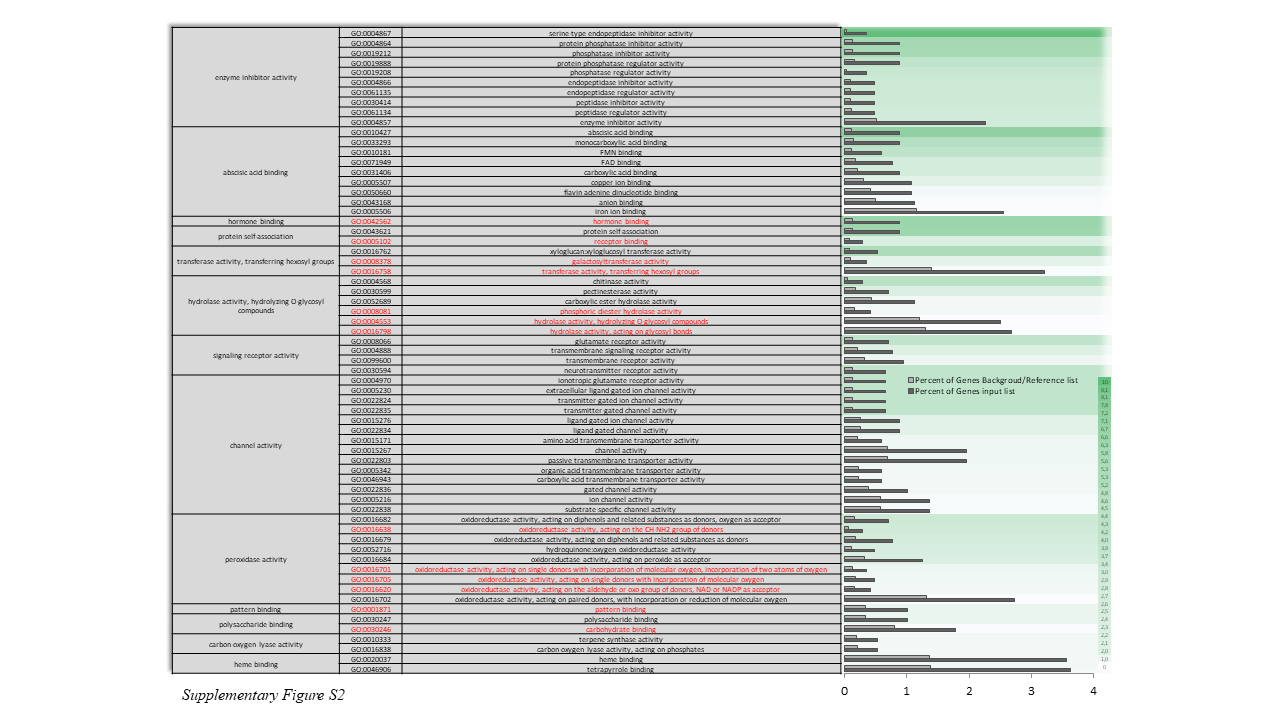

Supplement: SUPPLEMENTARY FIGURE S2 — Specific gene ontology enrichment. DEGs obtained between AH and NCS. [file Image_2.tif]

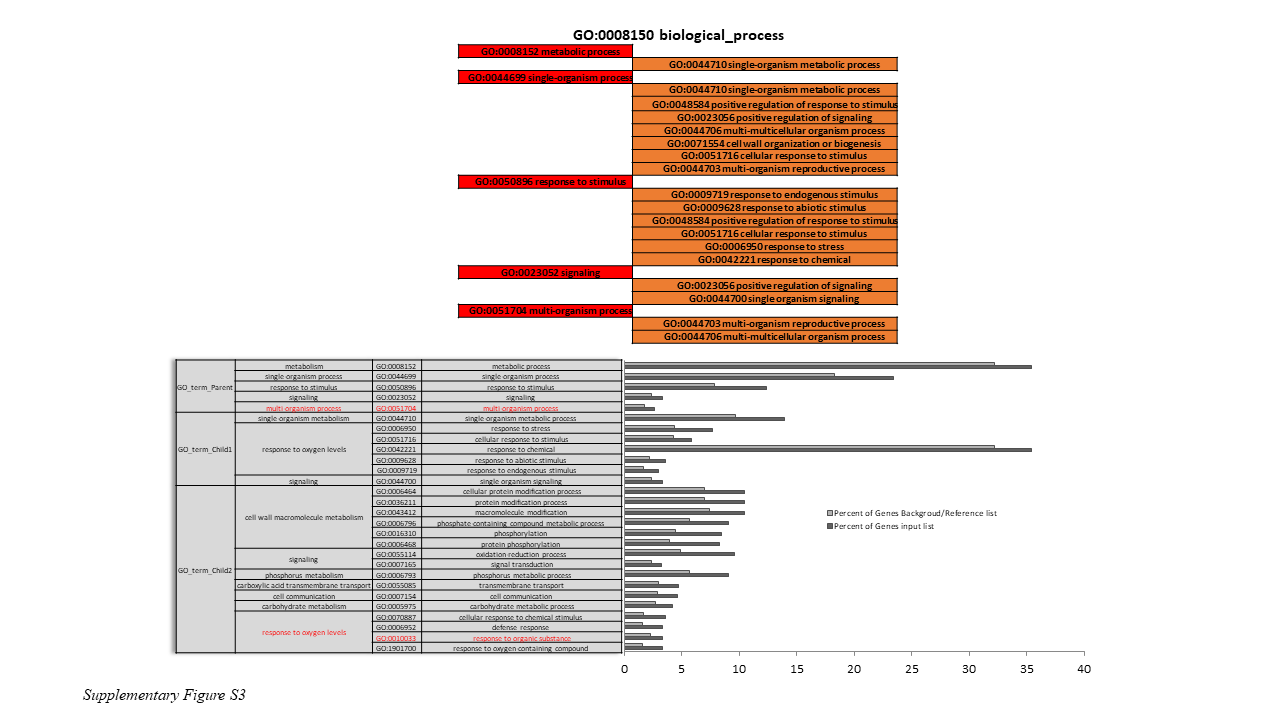

Supplement: SUPPLEMENTARY FIGURE S3 — Total gene ontology enrichment. DEGs obtained between AH and ACS. Total gene ontology enrichment. A Singular Enrichment Analysis (SEA; FDR ≤ 0.05) showing the GO for the most significant biological processes categories. [file Image_3.tif]

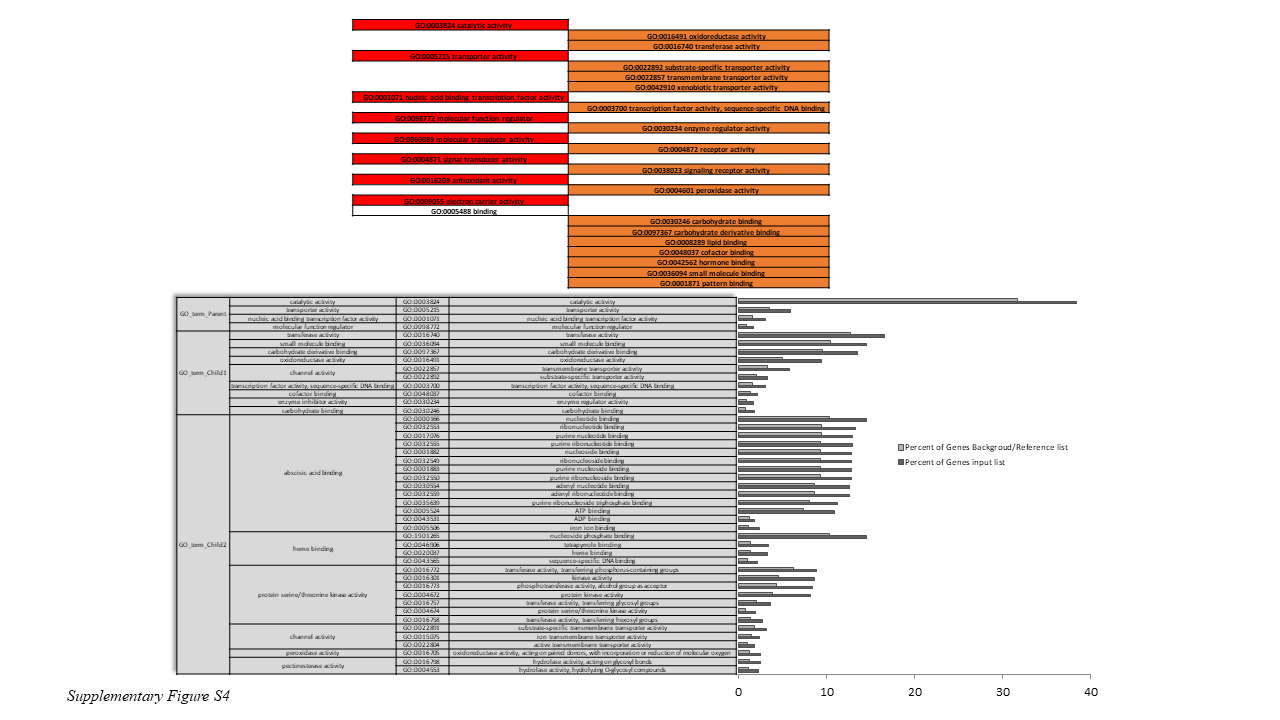

Supplement: SUPPLEMENTARY FIGURE S4 — Total gene ontology enrichment. DEGs obtained between AH and ACS. Bar chart showing in detail the most significant molecular function terms overrepresented obtaining by a Singular Enrichment Analysis (SEA; FDR ≤ 0.05). [file Image_4.tif]

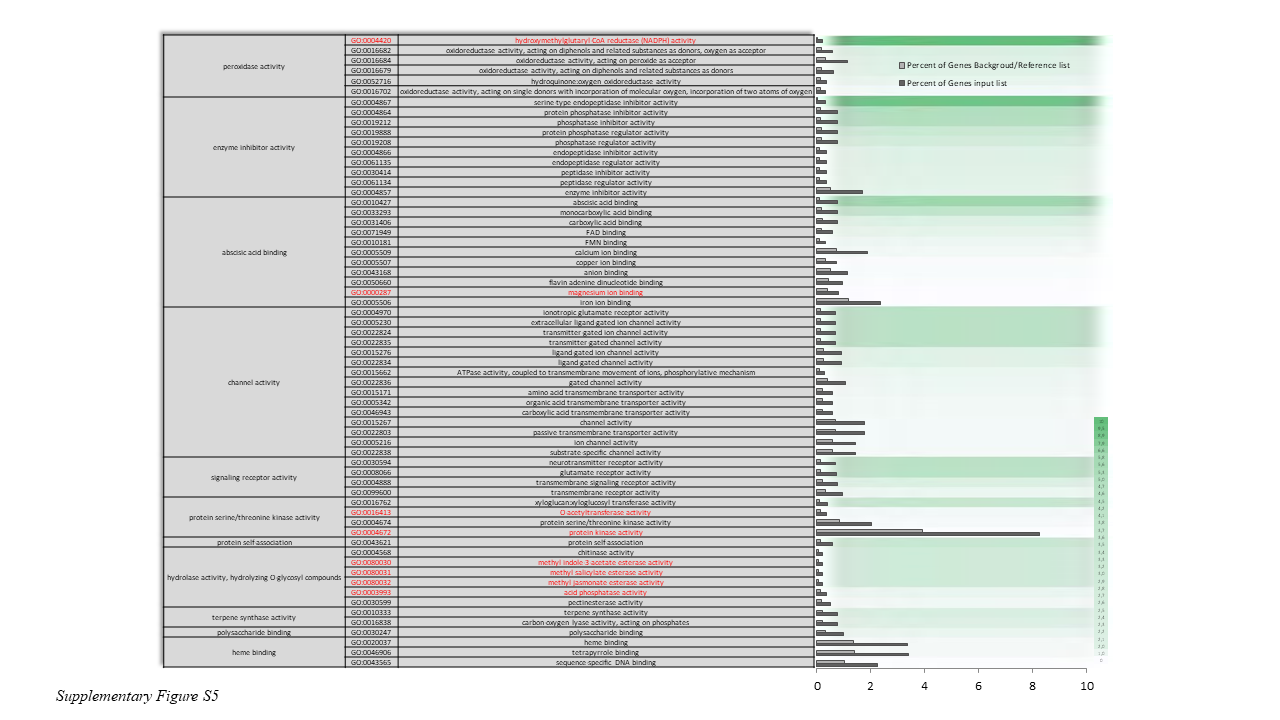

Supplement: SUPPLEMENTARY FIGURE S5 — Specific gene ontology enrichment. DEGs obtained between AH and ACS. [file Image_5.tif]

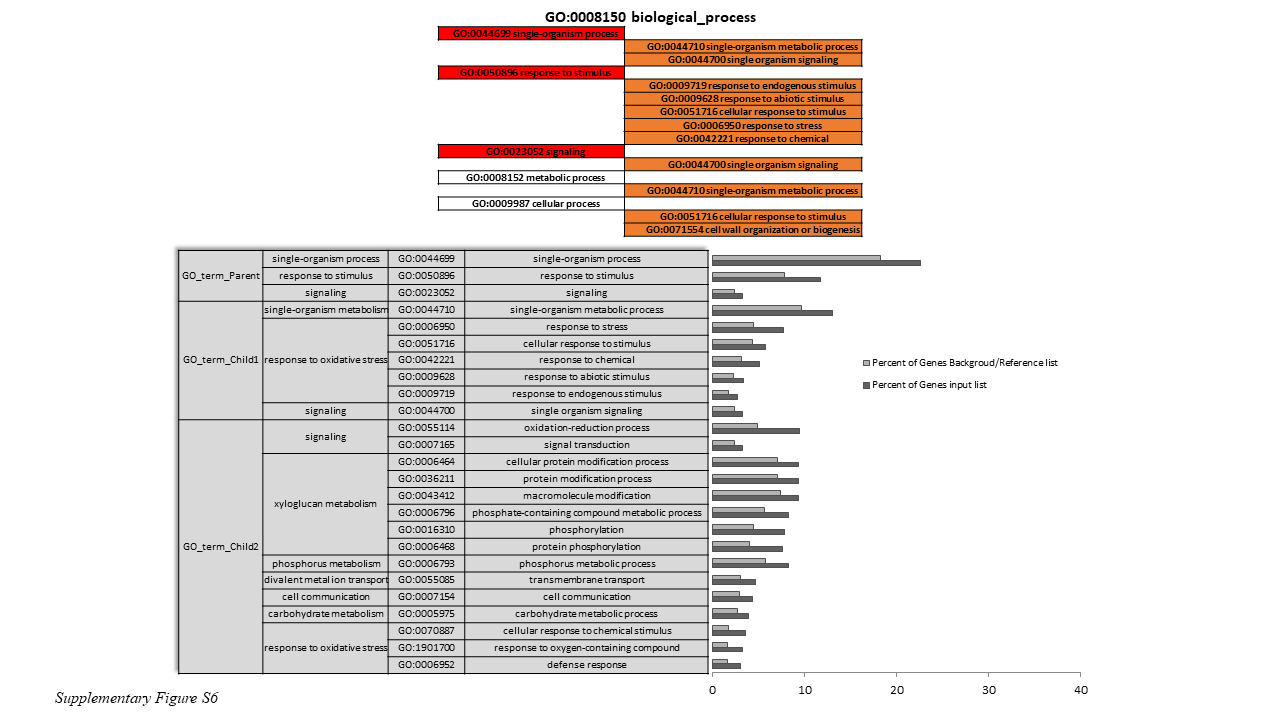

Supplement: SUPPLEMENTARY FIGURE S6 — Total gene ontology enrichment. DEGs obtained between AH and CCS. A Singular Enrichment Analysis (SEA; FDR ≤ 0.05) showing the GO for the most significant biological processes categories. [file Image_6.tif]

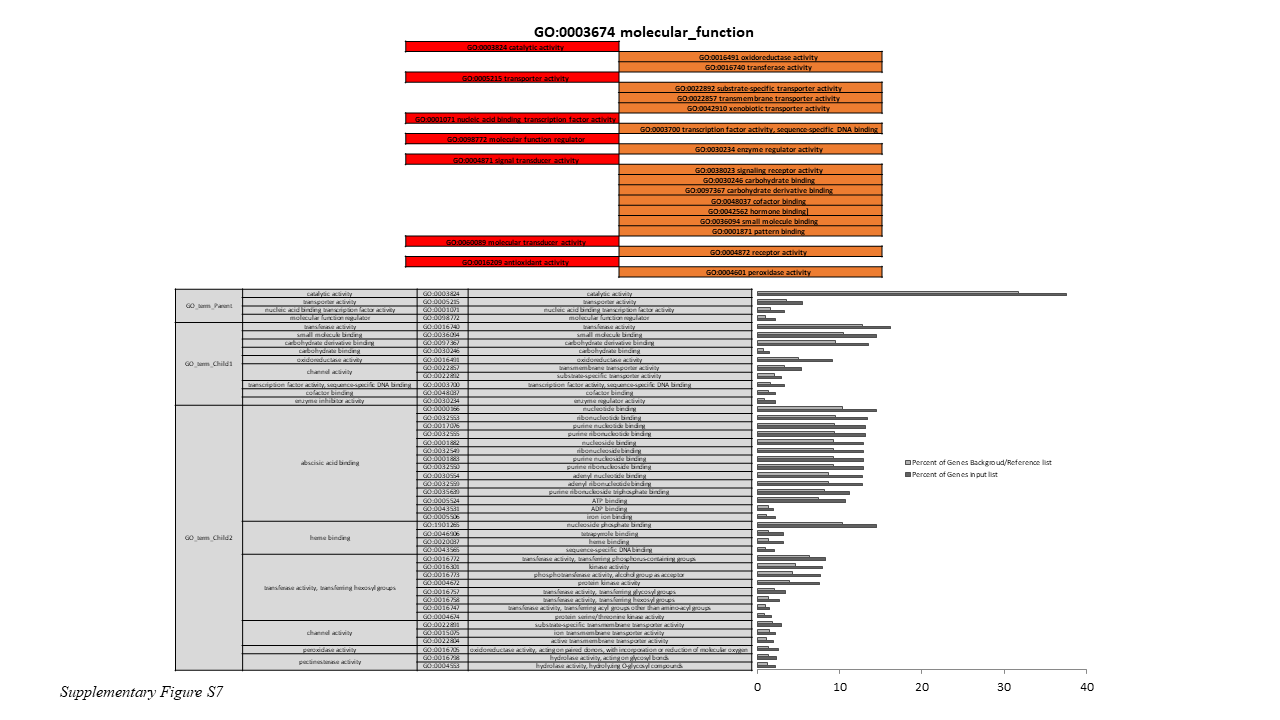

Supplement: SUPPLEMENTARY FIGURE S7 — Total gene ontology enrichment. DEGs obtained between AH and CCS. Bar chart showing in detail the most significant molecular function terms overrepresented obtaining by a Singular Enrichment Analysis (SEA; FDR ≤ 0.05). [file Image_7.tif]

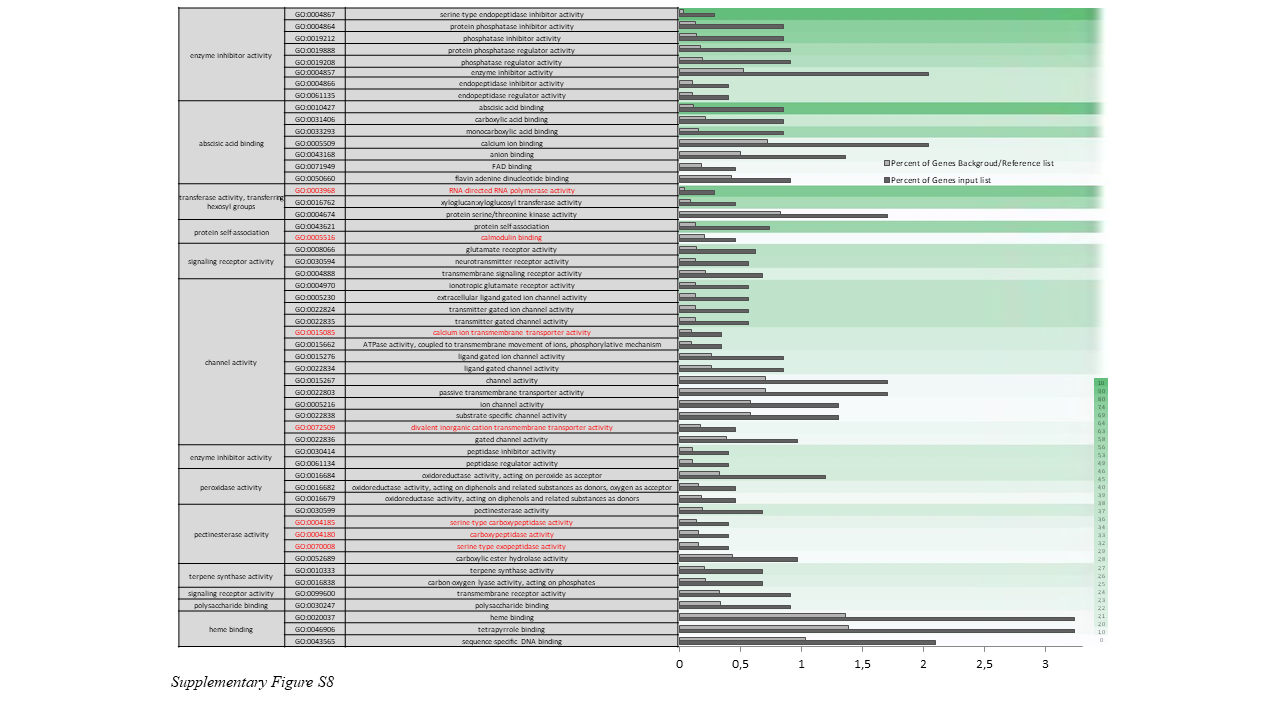

Supplement: SUPPLEMENTARY FIGURE S8 — Specific gene ontology enrichment. DEGs obtained between AH and CCS. [file Image_8.tif]

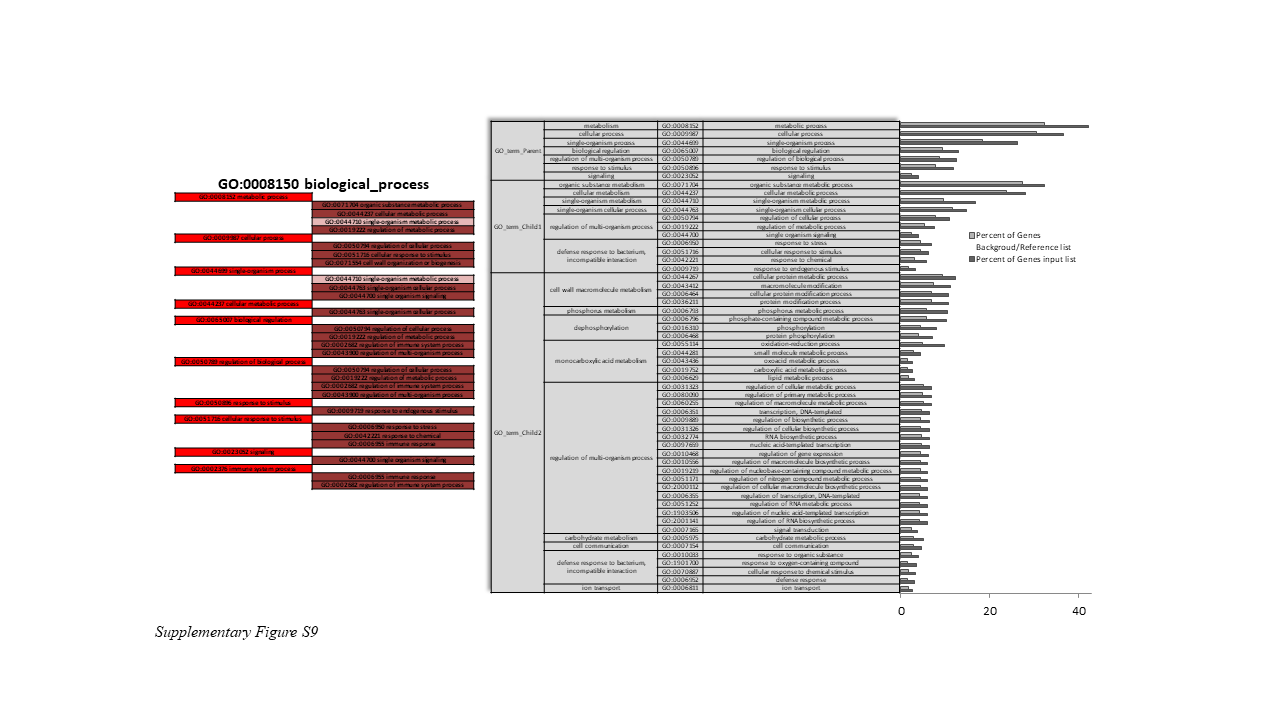

Supplement: SUPPLEMENTARY FIGURE S9 — Total gene ontology enrichment obtained for firmness using Weighted Gene Correlation Network Analysis. Bar chart showing in detail the most significant biological processes categories obtaining by a Singular Enrichment Analysis (SEA; FDR ≤ 0.05) in WGCNA. [file Image_9.tif]

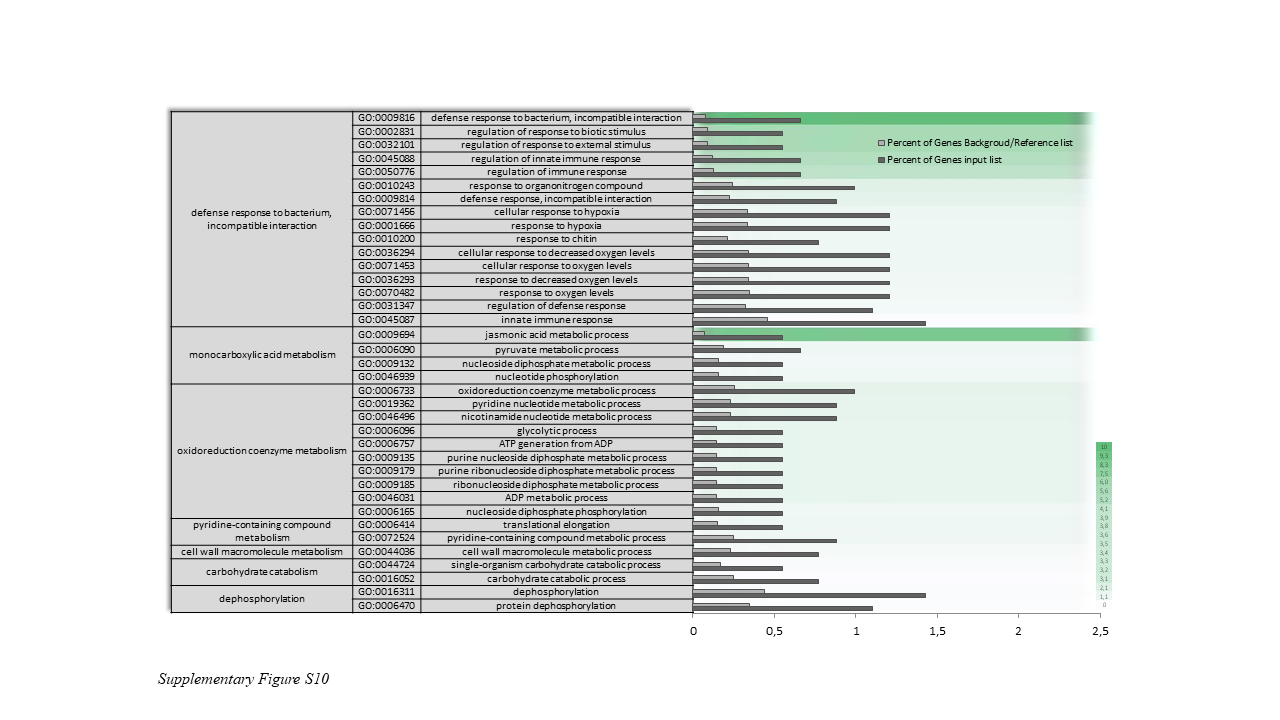

Supplement: SUPPLEMENTARY FIGURE S10 — Specific gene ontology enrichment obtained for firmness using Weighted Gene Correlation Network Analysis. [file Image_10.tif]

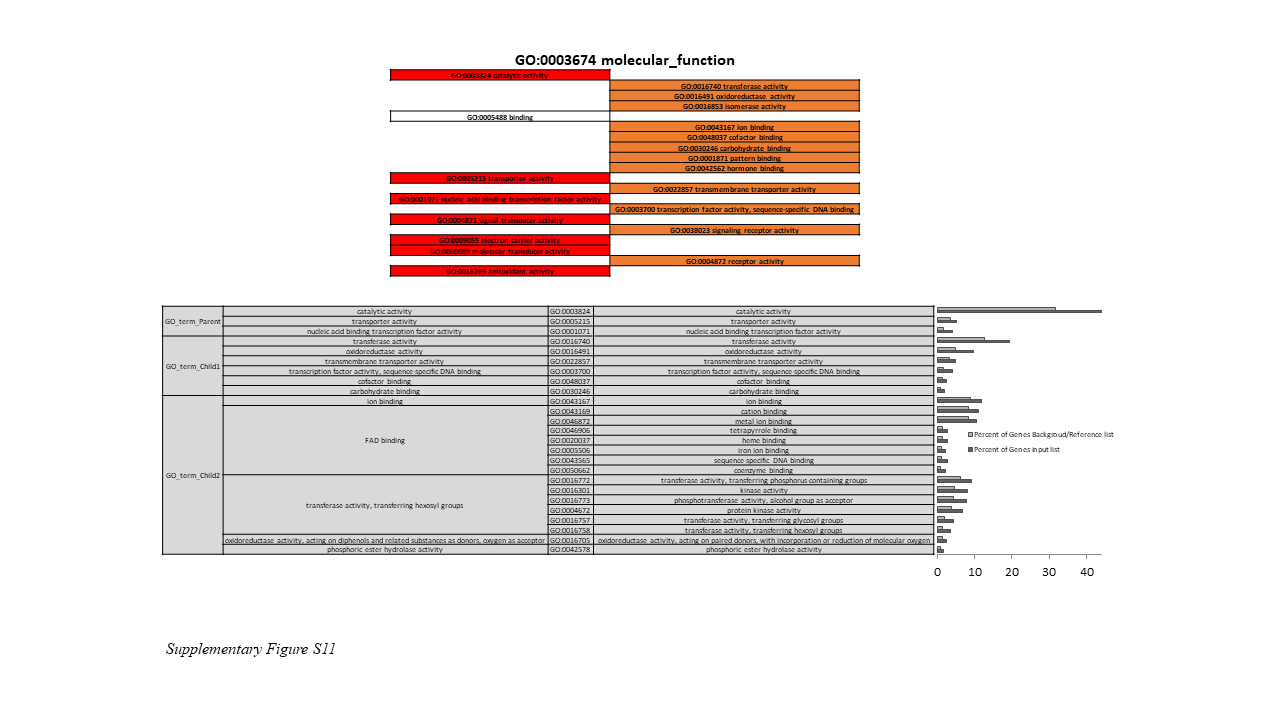

Supplement: SUPPLEMENTARY FIGURE S11 — Total gene ontology enrichment obtained for firmness using Weighted Gene Correlation Network Analysis. Bar chart showing in detail the most significant molecular function terms obtaining by a Singular Enrichment Analysis (SEA; FDR ≤ 0.05) in WGCNA. [file Image_11.tif]
