# Supplementary material for: Transcriptomic analysis of CO2-treated strawberries (Fragaria vesca) with enhanced resistance to softening and oxidative stress at consumption
Source: Front Plant Sci. 2022 Aug 19;13:983976. doi: 10.3389/fpls.2022.983976 (PMC9437593; doi:10.3389/fpls.2022.983976)
Supplement: Supplementary file 12 [file Table_1.DOCX]

| **NAME** | **SEQUENCE** | **Efficiency** | **AMPLICON SIZE (bp)** | **GENE** |
| --- | --- | --- | --- | --- |
| FvH4_1g02620_RAFF_F | TGGAGCCATTGAAGAGTTGA | 2,1 - 1,89 | 162 | *Raffinose synthase family protein FvH4_1g02620* |
| FvH4_1g02620_RAFF_R | AAAGACACCAACCCAGATGC |  |  |  |
| FvH4_1g20060_ARAB_F | TATCTCCTCCACACCGACCT | 2,1 - 1,89 | 145 | *Arabinogalactan protein 5-like FvH4_1g20060* |
| FvH4_1g20060_ARAB_R | GAAGAAGCTCCCCACCAAAG |  |  |  |
| FvH4_1g24380_LACC1_F | CCTCTTCGAAACACCATTGC | 2,1 - 1,89 | 190 | *Laccase/Diphenol oxidase family protein FvH4_1g24380* |
| FvH4_1g24380_LACC1_R | GCATATCTGGTGGTGGAGGTA |  |  |  |
| FvH4_2g00610_CEL_F | ACTTGGTTCTGCGGTTTGC | 2,1 - 1,89 | 173 | *Celulase 3/Endoglucanase 3 FvH4_2g00610* |
| FvH4_2g00610_CEL_R | CAAATTTGTTATCCCAGCTGAAG |  |  |  |
| FvH4_3g42900_CELSYN2_F | GTTTTGTGAAGGAAGCAATCAG | 2,1 - 1,89 | 153 | *cellulose synthase like E1 FvH4_3g42900* |
| FvH4_3g42900_CELSYN2_R | GCAACTTCCCCTTGTCCTTT |  |  |  |
| FvH4_2g12370_αGAL_F | GCTTGGGGCATCGATTATTTA | 2,1 - 1,89 | 145 | *Alpha-galactosidase 1 FvH4_2g12370* |
| FvH4_2g12370_αGAL_R | GGTGCATATCTCCCCACTCG |  |  |  |
| FvH4_3g01440_FUCOSYLT1_F | CATAGTAAGCCCGAGCCAAA | 2,1 - 1,89 | 155 | *O-fucosyltransferase family protein FvH4_3g01440* |
| FvH4_3g01440_FUCOSYLT1_R | ACCCGCTGCTGGTTCATC |  |  |  |
| FvH4_6g37210_FUCOSYLT2_F | TGCTTCCAGCAAAACTGAAA | 2,1 - 1,89 | 106 | *O-fucosyltransferase family protein FvH4_6g37210* |
| FvH4_6g37210_FUCOSYLT2_R | GGCCATATGGTTCTTCTTCG |  |  |  |
| FvH4_4g30950_FUCOSYLT3_F | TCCAATCGATGGAGCCTTGT | 2,1 - 1,89 | 105 | *Galactoside 2-alpha-L-fucosyltransferase FvH4_4g30950* |
| FvH4_4g30950_FUCOSYLT3_R | CCTCACAATGCCTTACATGTGG |  |  |  |
| FvH4_4g27370_MAN1_F | TCGAGTTGATAAGGTTCTCCAAG | 2,1 - 1,89 | 173 | *GDP-L-galactose phosphorylase 2 FvH4_4g27370* |
| FvH4_4g27370_MAN1_R | TGACATTGATCGCAACAACAT |  |  |  |
| FvH4_3g23310_MAN2_F | GGTTCTCCAACCTTTTGATGG | 2,1 - 1,89 | 163 | *Mannose-1-phosphate guanylyltransferase FvH4_3g23310* |
| FvH4_3g23310_MAN2_R | ACTAACATTGATCGCAACAACAC |  |  |  |
| FvH4_5g23180_XYLH_F | GAAGCTTGCCTACCTCAACG | 2,1 - 1,89 | 173 | *Xyloglucan endotransglucosylase/Hydrolase 30 FvH4_5g23180* |
| FvH4_5g23180_XYLH_R | TCTTGAACCGTGCCTTCTCT |  |  |  |
| FvH4_5g37950_GLUCAN_F | ATCTCAGGAATGGGGTCAAA | 2,1 - 1,89 | 115 | *Glucan 1,3-beta-glucosidase FvH4_5g37950* |
| FvH4_5g37950_GLUCAN_R | GTTGATGAGCTCAACTGCGTA |  |  |  |
| FvH4_6g12410_LACC2_F | CCCCGATAATCCTCCGGTGA | 2,1 - 1,89 | 164 | *Laccase 7 FvH4_6g12410* |
| FvH4_6g12410_LACC2_R | GGTGATTCTCGATCGCTAAAA |  |  |  |
| FvH4_6g12430_LACC3_F | GATTACCCGGCGACTTCTATAAT | 2,1 - 1,89 | 126 | *Laccase 7 FvH4_6g12430* |
| FvH4_6g12430_LACC3_R | CTATGCCGAAGAAGAGTTGGTT |  |  |  |
| FvH4_4g20790_GALSYN_F | GGTGAAGAAATGGTGGGAGATA | 2,1 - 1,89 | 157 | *Galactinol synthase 2 FvH4_4g20790* |
| FvH4_4g20790_GALSYN_R | CGGTGCACTTCTCTGCTGAT |  |  |  |
| FvH4_6g22810_POLYGAL_F | TCCCCAGCTCGCTTTCTAT | 2,1 - 1,89 | 158 | *Polygalacturonase inhibitor FvH4_6g22810* |
| FvH4_6g22810_POLYGAL_R | GCCTCTGAAGTCCATCTTGC |  |  |  |
| FvH4_3g36410_EXPA4_F | CCAGGACTGGCTGGATGAG | 2,1 - 1,89 | 175 | *Expansin A4 FvH4_3g36410* |
| FvH4_3g36410_EXPA4_R | TTCTTTCCGGTGAAAGTCTGA |  |  |  |
| FvH4_1g00290_PLY_F | CGGGTCATGGAATTAGCATC | 2,1 - 1,89 | 197 | *Polygalacturonase/Pectin lyase family protein FvH4_1g00290* |
| FvH4_1g00290_PLY_R | GACCGGATTTTGAACGTTGT |  |  |  |
| FvH4_1g12210_FER_F | GATGCAGCTAGGGCTTTACG | 2,1 - 1,89 | 176 | *Ferulic acid 5-hydroxylase 1 FvH4_1g12210* |
| FvH4_1g12210_FER_R | GGCACACGACCCTTTTAGTC |  |  |  |
| FvH4_1g19194_RHA_F | TCGAACCTCCAAGAACTGGT | 2,1 - 1,89 | 150 | *Rhamnogalacturonate lyase family protein FvH4_1g19194* |
| FvH4_1g19194_RHA_R | ACAACCCGTACTGCCTGAAG |  |  |  |
| FvH4_2g02090_NINV_F | AAGACAACTGGCCCGAATAC | 2,1 - 1,89 | 179 | *Plant neutral invertase family protein FvH4_2g02090* |
| FvH4_2g02090_NINV_R | CCTCATTGCAGGCTTCATC |  |  |  |
| FvH4_2g25960_PME_F | AAGCAAATCGCGAAAGGTTTAT | 2,1 - 1,89 | 159 | *Plant invertase/Pectin methylesterase 41 FvH4_2g25960* |
| FvH4_2g25960_PME_R | GCGGCAGAGTTATTCGGAGT |  |  |  |
| FvH4_5g35580_PME_F | CAGACAATCATCACCGGAAG | 2,1 - 1,89 | 155 | *Plant invertase/Pectin methylesterase 20 FvH4_5g35580* |
| FvH4_5g35580_PME_R | TTTCGAACTGCAACTGCTTG |  |  |  |
| FvH4_5g35590_PME_F | CAGAAGGGTTTGTGGGAGTC | 2,1 - 1,89 | 154 | *Plant invertase/Pectin methylesterase 20 FvH4_5g35590* |
| FvH4_5g35590_PME_R | CGGAGAGAATGCGCGTATAG |  |  |  |
| FvH4_7g11320_PME_F | GTGACCGAGGCAATAACTACC | 2,1 - 1,89 | 131 | *Plant invertase/Pectin methylesterase 17 FvH4_7g11320* |
| FvH4_7g11320_PME_R | ATGCCATCTCCCATCAACATA |  |  |  |
| FvH4_2g39441_JAS_F | TGCGTACTCACTTCAATTCCAC | 2,1 - 1,89 | 178 | *Jasmionic acid carboxyl methyltransferase FvH4_2g39441* |
| FvH4_2g39441_JAS_R | AACCATGCTCATCAATGCAA |  |  |  |
| FvH4_5g20290_EXP_F | GGACACTCAAAGCCCACCT | 2,1 - 1,89 | 142 | *Expansin-like B1 FvH4_5g20290* |
| FvH4_5g20290_EXP_R | GAGCTGAATGTCTGATGTATAGGC |  |  |  |
| FvH4_5g21120_EXP_F | TCAGCAGCCTCTGACCTCTTT | 2,1 - 1,89 | 159 | *Expansin-like B1 FvH4_5g21120* |
| FvH4_5g21120_EXP_R | CGCTCGTTGGCTGAGGATA |  |  |  |
| FvH4_6g38170_XYL_F | AAGCCAATGCTTGTCTTGC | 2,1 - 1,89 | 176 | *Xyloglucan endotransglucosylase/Hydrolase 15 FvH4_6g38170* |
| FvH4_6g38170_XYL_R | ACCTTGCGGGAATCGTTTA |  |  |  |
| FvH4_6g38980_CAT_F | CGAGTGATGCCGAGGTTATT | 2,1 - 1,89 | 195 | *MATE efflux family protein FvH4_6g38980* |
| FvH4_6g38980_CAT_R | TTTTGAAGGCAAGAACACACC |  |  |  |
| FvH4_3g40160_PER_F | AAACAAAACTGCCCACGAAC | 2,1 - 1,89 | 153 | *Peroxidase superfamily protein FvH4_3g40160* |
| FvH4_3g40160_PER_R | GTCACTTCCATCGCCTTTG |  |  |  |
| FvACT_F | GGGTTTGCTGGAGATGATG | 2,1 - 1,89 | 292 | *ACTIN FvH4_7g22410* |
| FvACT_R | CACGATTGGCCTTGGGATTC |  |  |  |

**Table S1.** List of primer sequences used in this study including the efficiency and the amplicon size.
